# Words by the tail: Assessing lexical diversity in scholarly titles using frequency-rank distribution tail fits

**DOI:** 10.1371/journal.pone.0197775

**Published:** 2018-07-09

**Authors:** Nicolas Bérubé, Maxime Sainte-Marie, Philippe Mongeon, Vincent Larivière

**Affiliations:** 1 École de Bibliothéconomie et des Sciences de l’information, Université de Montréal, C.P. 6128, Succ. Centre-Ville, Montréal, QC. H3C 3J7, Canada; 2 Centre for Science and Technology Studies, Leiden University, P.O. Box 905, 2300 AX Leiden, The Netherlands; 3 Observatoire des Sciences et des Technologies (OST), Centre Interuniversitaire de Recherche sur la Science et la Technologie (CIRST), Université du Québec à Montréal, CP 8888, Succ. Centre-Ville, Montréal, QC. H3C 3P8, Canada; KU Leuven, BELGIUM

## Abstract

This research assesses the evolution of lexical diversity in scholarly titles using a new indicator based on zipfian frequency-rank distribution tail fits. At the operational level, while both head and tail fits of zipfian word distributions are more independent of corpus size than other lexical diversity indicators, the latter however neatly outperforms the former in that regard. This benchmark-setting performance of zipfian distribution tails proves extremely handy in distinguishing actual patterns in lexical diversity from the statistical noise generated by other indicators due to corpus size fluctuations. From an empirical perspective, analysis of Web of Science (WoS) article titles from 1975 to 2014 shows that the lexical concentration of scholarly titles in Natural Sciences & Engineering (NSE) and Social Sciences & Humanities (SSH) articles increases by a little less than 8% over the whole period. With the exception of the lexically concentrated Mathematics, Earth & Space, and Physics, NSE article titles all increased in lexical concentration, suggesting a probable convergence of concentration levels in the near future. As regards to SSH disciplines, aggregation effects observed at the disciplinary group level suggests that, behind the stable concentration levels of SSH disciplines, a cross-disciplinary homogenization of the highest word frequency ranks may be at work. Overall, these trends suggest a progressive standardization of title wording in scientific article titles, as article titles get written using an increasingly restricted and cross-disciplinary set of words.

## Introduction

From a historical and etymological point of view, article titles are closely linked to roman *tituli*, meaning ‘inscriptions’ or ‘marks’ and referring to the labels hanging from the extremity of scrolls [1]. By allowing for author or content identification without any prior unscrolling of documents, such *tituli* are functionally evocative of modern scholarly article titles, as the latter represent the first and increasingly only point of contact between scholars, their work, and the scholarly community [2–15]. Indeed, due notably to the exponential growth in scholarly production [16–21], an increasing number of scholars “are content with reading the title only of the papers they deem interesting for their research purposes.” [13] Evidence even shows that medical doctors sometimes make clinical decisions on the sole basis of title content [22, 23].

These research practices are closely related to the evolution of title writing in the 20th century, characterized by an increased focus on both informativeness and attractiveness [24, 25]. On the one hand, scholarly titles have progressively been endowed with the task of clearly, concisely, and accurately reflecting article content [26–31], especially “by describing its methods, design, results or conclusion, or by revealing important contextual attributes.” [32] On the other hand, titles play an increasing role as “attention triggers,” [33, 34] attracting readership and increasing both article visibility and scholarly impact [35].

Both tendencies have not only increased the importance and significance of scholarly titles, but also contributed in making them objects of scientific investigation. As testimony to this, the number of recent studies thereon has not only grown, but also fueled the use of the term “titleology.” [13–15, 36–47] In particluar, linguistic analysis of titles in intra-disciplinary [9, 48, 49], interdisciplinary [5, 10, 24, 29, 50–54], and intercultural [55, 56] context as well as between academic genres [30] have shown that, regarding syntax and surface characteristics, scholarly titles “vary and, at the time, display similarities across a number of factors and in several dimensions, such as structure, syntactic encoding, length, wording, use of punctuation marks, informativeness, functions, and information sequencing.” [13]

In parallel, various studies have looked onto title wording as a means to gain insights regarding the “processes of discourse formation” [57] within disciplines and in the scholarly community in general [34, 57–64]. Indeed, though their research activities, scholars contribute to the formation of various disciplinary languages, processes that collectively shape the scholarly discourse [65] and whereof titles can give a short, condensed but adequate portait [63]. In particular, the frequency analysis of title words has proven to be “a powerful approach to analyze the… development of scientific fields.” [57] It has indeed been shown that terms that rise and decline in frequency tend to be associated with topical issues or terminologies [64, 66]. A good example is the use of the word “tax” in economy, which became the second-most popular substantial title term in the 50s but quickly declined afterwards [67]. A similar rise-and-fall tendency has also been observed for research-related clichés in medical article titles (e.g. “paradigm shift”, “out of the box”) [68]. In the case of social sciences, a comparison of word frequencies within article titles in history, sociology, economics, and education found history to use rarer terms, which often referred to people or place names [69].

Little research has however been devoted to the lexical diversity of scholarly titles. This research gap is rather surprising, since the question as to whether scholarly discourse evolves towards a more disparate or concentrated vocabulary is of paramount relevance and importance. At first glance, both hypotheses seem reasonable. On the one hand, the growth of scholarly literature, reflected notably in the increasing number of publication venues [70–73], institutions, and researchers [74–76], suggests “that the extent of the cognitive territory of science must be expanding,” [63] expansion which should cause a diversification of scholarly vocabulary. On the other hand, given the fact that one of the functions of disciplinary languages is “to ensure effective transmission of knowledge by stabilizing the vocabulary,” [65], one might suppose that such stabilization should lead to an increase in concentration of the scholarly lexicon:

“If different researchers work on the same set of subject-related research problems and concepts, one would expect that they use, to a relatively large extent, the same words for important concepts and problems in their specialty” [77].

To our knowledge, only one previous study addresses that specific question [63]. Using the number of distinct noun phrases in scientific titles as a proxy for the conceptual breadth of the articles they entitle, the author tracks the evolution of vocabulary diversity in physics, astronomy, and biomedicine. Following the extraction of noun phrases from the article titles of each discipline and annual randomization of article order, this study proceeds by calculating the number of different noun phrases within consecutive segments of 1,500 noun phrases. Results for all three disciplines show that while the publication volume increases from a hundred- to a thousand-fold, the number of distinct noun phrases grows “within a factor of few”, and the fact that this rise is related but not identical to the increase in publication volume leads the author to conclude in the “cumulative nature of science.” [63]

While this research constitutes a worthwhile contribution to the advancement of knowledge, some methodological choices made in order to avoid corpus size dependency effects are however debatable. Research in statistical linguistics has indeed shown that the use of fixed size sampling techniques raises some issues as regards to the representativity or reliability of the results obtained. These concerns are all the more relevant given the concomitant-but-not-identical growth of the noun phrase lexicon and the publication volume, which hints at the possibility of sample size effects still being at work. In light of these caveats, the purpose of the present research is to assess the evolution of lexical diversity in scholarly article titles using a new indicator of lexical diversity based on zipfian frequency-rank distribution tail fits. The outline of the article is as follows: following a general presentation of lexical diversity measurement, the methodological steps leading to the design, test, and comparative evaluation of our indicator as regards to corpus size dependency are then presented. Finally, this lexical indicator is used to assess the evolution of lexical concentration in scholarly article title wording on a disciplinary basis, followed by concluding remarks and general thoughts on the prevalence and origin of zipfian frequency-rank distributions in language.

## Background: The measurement of lexical diversity

Used more or less interchangeably with the terms *lexical variation, lexical variety, lexical variability*, and *lexical flexibility* [78–80], the term *lexical diversity* is here defined as the extent of vocabulary disparity within a given language sample. Conceived in this way, lexical diversity is the exact opposite of lexical concentration: the higher the proportion of frequent words within a language sample is, the more unbalanced the vocabulary use is, and thus the higher and the lower lexical concentration and diversity respectively are.

Within the linguistic community, the term lexical diversity is usually preferred, as it is deemed indicative of vocabulary quality and linguistic proficiency: “there is a general underlying assumption… that a high lexical diversity is ‘a good thing’, an indication of a combination of vocabulary size and the ability to use it effectively.” [81] In fact, research show that when listeners can fully understand a speaker’s message, their perception of the latter’s credibility, competence, likability, socio-economic status, and communicative effectiveness is positively correlated to the diversity of his vocabulary [82].

Operationally, assessing lexical diversity revolves around measuring “the proportion of words in a language sample that are not repetitions of words already encountered.” [83] To that end, the Peirce-inspired distinction between type and token counts is often invoked [84]: the token count of a language sample represents “the total number of words it contains,” while the type count consists in “the number of different words in it.” [81] Take for example the following sentence:

$$\begin{array}{c} \text{A}\;\text{horse}\;\text{is}\;\text{a}\;\text{horse,}\;\text{of}\;\text{course,}\;\text{of}\;\text{course.} \end{array}$$

In this sentence, which consists of 9 word tokens, 5 different word types are to be found: ‘a’, ‘horse’, ‘is’, of’, ‘course’. With the exception for ‘is’, which occurs only once in that sentence, each word type is thus instantiated by two word tokens. It is precisely this imbalance between token and type counts that allows for the quantification of lexical diversity: the higher the level of word repetition in a language sample, the greater the imbalance, and thus the lower the former’s lexical diversity.

The Type-Token Ratio (henceforth TTR) represents the most used and intuitive way to measure lexical diversity on the basis of word repetition patterns. Allegedly proposed for the first time by Johnson [79, 85], the TTR consists in expressing the number of different words “as a proportion of the total number of words.” [81]

$$\begin{array}{c} \text{TTR}=\text{Type}\;\text{Count}/\text{Token}\;\text{Count} \end{array}$$ (1)

Thus, the higher the probability that a new word token is also a new word type, the closer the TTR is to 1, and the greater the lexical diversity of that text. Returning to the above example, the sentence contains 9 word tokens and 5 different word types (’a’, ‘horse’, ‘is’, ‘of’, ‘course’), which means that its TTR score is 5/9 = 0.56.

At first glance, ratios such as the TTR allow for better comparability and measurement than single quantities like token counts. However, it must be stressed that this is only true insofar as the relationship between the quantities being compared is proportional or linear [81]. Unfortunately, the relationship between type and token counts within the context of language production does not meet this requirement: since each new word within a language sample automatically increments the token count by 1 but only increases the type count by 1 if that word wasn’t already present in the sample, the type count thus increases “at a slower rate than the token count” [81], and that rate keeps getting slower as the probability of word repetition grows with each new word.

As regards to the TTR, this property implies that the values returned by the indicator inevitably falls towards zero with increasing language sample size [86]. What is called the “sample size problem” in lexical diversity research can thus be stated as follows: given that the probability of a new word being used in the text decreases as the latter grows longer, lexical diversity ultimately depends on text length, which means that any attempt to measure lexical diversity based on “the proportion of repeated words in a language sample” [87] has to properly control for sample size on word repetition rates.

### Lexical diversity and sample size

Sample size dependency issues are not unique to the TTR nor the measurement of lexical diversity at large. On the contrary, all linguistic investigations dealing with repetition patterns in language must properly control for sample size.

This property sets lexical statistics apart from most other areas in statistics, where an increase in the sample size leads to enhanced accuracy and not to systematic changes in basic measures and parameters [88].

In the case of lexical diversity measurement, a common strategy used to cope with sample size dependency consists in finding an adequate mathematical expression of the type count slowdown in order to counterbalance its effect on the TTR. Various attempts were made in this regard: some studies [89, 90] assumed that the ratio fall is proportional to the square root of the token count and attempted to cushion the former accordingly, while others have tried to ‘linearize’ the same ratio fall through various logarithmic transformations [91–95]. However, far from solving the sample size problem, these different attempts “merely change the shape of the curve or alter the scale.” [81]

In fact, the only strategy that has so far successfully dealt with the sample size dependency of the TTR or any TTR-based measure consists in controlling for sample upsizing through fixed size sampling procedures. In this regard, two lexical diversity indicators have shown to be relatively constant across wide ranges of sample sizes, namely the Mean Segmental Type-Token Ratio (MSTTR) [79, 85] and the Measure of Textual Lexical Diversity (MTLD) [81, 96, 97].

These two measures are essentially mirror images of each other, MSTTR holds the sample size constant while calculating the mean TTR across different segments of a text, whereas MTLD holds TTR constant (usually at.72) while calculating the average number of words in any segment of text that remains above the TTR cutoff value [83].

Similar in kind to the method advocated in the above-mentioned study of noun phrase diversity in scientific titles [63], such fixed size sampling procedures are not without flaws, however. First and foremost, none of these measures “evaluates the text as a unified whole,” [83] which raises the possibility of results obtained being an artefact of sampling.

Consider for example a text consisting of four paragraphs of equal length, with each paragraph having an equivalently high TTR. Depending on how MSTTR and MTLD segment the text, both indices are likely to show that the text has a high overall TTR value even if the last three paragraphs in the text are exact copies of the first paragraph [83].

Moreover, different sampling standardization methods have been shown to lead to widely diverging results, which “makes it difficult to compare the results from studies which use different standardisation procedures.” [81] Given that there is still no universally agreed way of standardizing samples [81], this suggests that fixed size sampling techniques do not solve the sample size dependency problem at all, but merely transform it into a sampling scheme dependency problem.

While these shortcomings seems to put into question the relevance of word repetition rates in language samples “as a reliable measure of diversity,” [81] lexical diversity measures based on type and token counts have nevertheless led to interesting experimental and clinical results. Indeed, in cases where language samples are of relatively small size, “empirical studies have found lexical diversity variables to be valid as developmental indices and as theoretically motivated measures in profiling a range of language disabilities.” [81]

Research and diagnostic applications of lexical diversity can be found in a wide range of linguistic fields, including first and second language acquisition, linguistic input and interaction, demographic influences on language performance, language impairment and delay, aphasia, schizophrenia, stylistics, forensic linguistics, and many more [98].

However, from a mathematical standpoint, the sample size problem remains a challenge, and the need to control for token counts becomes more problematic as language samples grow. In order to overcome this stalemate, mathematically more sophisticated attempts were proposed over the years. Amongst this group of models, those based on frequency-rank distributions certainly stand out in both quantity and impact. The following section gives a brief historical review of these models, followed by a critical assessment of their use for lexical diversity measurement.

### Lexical diversity and frequency-rank distributions

Broadly defined, frequency distributions are organized tabulation/graphical representations of the number of times a given value occurs in a data set. In the case of linguistic corpora, each entry in the distribution refers to a unique word type and its value is defined by its token count, that is, the number of times it occurs in the corpus. Frequency-rank distributions of words represent a special kind of lexical distribution, in which all word types are ordered and ranked “as a series of decreasing frequencies.” [99] In this way, corpora are thus strictly modelled in terms of cardinal and ordinal information: each word type is reduced to an ordered pair of numbers representing to its token count and its rank in the decreasing frequency distribution.

The origin of word frequency-rank distributions can be traced back to the work of Jean-Baptiste Estoup. Estoup was a specialist, eulogist, and teacher of the Duployan system, a shorthand method based on a simple geometric phonetic alphabet, initially designed for the education of illiterate people. Since “the prime motivation for shorthand has always been the ability to transcribe speech verbatim at high speed,” [100] myriads of subsets of abbreviating symbols referring to common expressions used in different professional settings [101] were grafted into the phonetic alphabet system. Some stenographers from Estoup’s time even proposed to supplement the Duployan system with subsystems of simpler and easier-to-plot symbols for the most frequent words [102]. Radically opposed to the latter and actively committed to the development of a single and unified shorthand system, Estoup advocated a scientific and evidence-based stenographic approach, based on rational rules and empirical evidence. The theoretical outline of his book *Gammes sténographiques* [103], included if not originally, then from the third edition onward, is exemplary in this regard: in order for learners to become speech-quick stenographers as early as possible, Estoup extracted a word frequency distribution from speeches of various oratory and epistolary styles in order to measure, for each successive segment of 1,000 words, the number of different words (type count), their frequency (token count) in descending order, and their average rate of repetitiveness (equivalent to the TTR).

From a stenographical and pedagogical perspective, the rationale behind this procedure was sound: since an improvising speaker whose speech a stenographer has to transcribe only has a limited range of words at his disposal, repetition is bound to occur, and the fastest way to become an efficient stenographer is to find which words are the most likely to occur, then learn and practice their stenographic transcription [103]. From a statistical linguistic perspective, Estoup’s contributions are foundational: by generating the first known word frequency-rank distributions, tables, and charts, the author was able to share unprecedented observations regarding “the hyperbolic nature of the frequency of word usage,” [104] according to which a few high-frequency word types account for the vast majority of tokens in a language sample.

Estoup’s methodological and empirical insights on hyperbolic frequency-rank word distributions were furthered independently by the nuclear physicist Edward Uhler Condon and the American linguist George Herbert Zipf. Through empirical and visual analysis of various language samples, both researchers found out that the relationship between the frequency rank of word types and their token count is constant over whole distributions and follows an exponentially decreasing function [105, 106]. The role played by mathematical visualization in this discovery, notably through the use of what seems to be the first Pareto-like charts of statistical linguistics, can hardly be overestimated: by plotting word type rankings and frequencies on logarithmic scales, both authors are able to show that the inverse relationship between both variables is surprisingly regular. Based on the slope-intercept form for linear Equations (*y* = *mx* + *b*), these mathematical visualization experiences undertaken by both authors can be algebraically expressed as the linear Equation

$$\begin{array}{c} \log O(R)=-s\log R+\log c \end{array}$$ (2)

where *R* corresponds to the rank of a word type in the frequency-rank lexical distribution of a corpus, *O*(*R*) to the token count of that word type (*O* here stands for ‘occurrences’), −*s* to the negative direction and steepness of the slope, and *c* the vertical intercept of the latter, which varies according to the length of the corpus. By converting Eq (2) to non-logarithmic form, the standard and current formulation of Zipf’s famous law is obtained:

$$\begin{array}{c} O\left(R\right)=\frac{c}{R^{s}} \end{array}$$ (3)

Eq (3) is of the utmost importance for the research on lexical diversity. Indeed, the diversity of the lexical distribution of a given language sample is here given by *s*, which corresponds to the steepness of the slope in the logarithmic form of the distribution given in Eq (2): the higher the value of *s*, the steeper the slope, and thus the lower the lexical diversity and the higher the lexical concentration. However, both Condon and Zipf didn’t pay much attention to this exponential variable in their analysis of lexical distributions: Condon simply estimated the value of *s* using an logarithmic approximation of the *c*th harmonic number based on the Euler-Mascheroni constant, focusing instead on solving the Equation for *c*, which corresponds to the y-intercept of the linear function in Eq (2) [105]. In Zipf’s case, the linear curve fitting done in log-log plots were apparently made “by visual judgment only” [107]: “finding their slopes to be ordinarily close to -1, he appears to have assumed that the “true” slope of such curves was -1.” [107] This questionable assumption has led to the simplistic but often used form of Zipf’s law, which states that for any corpus, “the frequency of a given word multiplied by its rank produces a number which is roughly the same as the product of rank and frequency of any other word in the corpus.” [108]

$$\begin{array}{c} O\left(R\right)=\frac{c}{R} \end{array}$$ (4)

Despite these misinterpretations, the use of frequency-rank distribution slopes as indicators of lexical diversity was not too long in coming. In an attempt to test the rule on word lists produced by 1,000 normal subjects in a word association test of 1.00e2 stimulus words, Skinner found that setting *s* to 1.29 and *c* to 300 provided the best fit for the collected data [109]. However, the author noted that the rule did not apply well to the first few ranks [109]. A year later, the psychologist John Carroll used an approach similar to that of Condon, setting the value of *s* through harmonic number approximation in order to find the value of *c*, which the author considers as indicative of lexical diversity. Testing his formula on language samples extracted from word production tasks and various literary texts, Carroll found out that neither the repetition rate nor the relationship between ranks and frequencies proposed by Zipf remain constant across differing sample sizes [110]. Along the same lines, John W. Chotlos attempted to find the value of *s* and *c* for 18 language samples drawn at random from 108 transcripts of children’s writing. In the end, Chotlos found out that *s* had a mean value of 0.845 for all samples, but that this mean value rose to 0.974 when the 20 best-ranked word types are removed from all distributions. In both distributions types however, the range of values for *s* is rather large, spanning from 0.796 to 0.938 and 0.808 to 1.255 respectively. Thus, in these cases as in earlier studies, high-ranked word types seem to follow a different regime than the other, less frequent, word types. As for *c*, the author computed its value through successively longer and longer sequential sub-samples and concluded that it was subject to sample size. On the basis of these results, Chotlos concluded that the functional representation of the relationship between word types and tokens “is of no simple nature.” [111]

Perhaps the most significant contributions in this matter are those made by Bruno Mandelbrot within the theoretical framework of ‘macrolinguistics’, a term he coined to refer to “the description and study, by statistical means, of large-scale linguistic phenomena”. [112] In various studies of Zipf’s law, Mandelbrot investigated whether the value of *s* is effectively −1 and whether the product of *R* and *O*(*R*) is indeed equal to *c* [107, 113]. Discovering that *s* does indeed vary, Mandelbrot proposed and derived as early as 1951 [114, 115] a generalization of Zipf’s law that more closely fits lexical distributions in language samples, generalization which consisted in shifting the rank value by an amount *β*” [116] and resulted in what is often known as the “Canonical Law”:

$$\begin{array}{c} O\left(R\right)=\frac{c}{{\left(R+\beta \right)}^{s}} \end{array}$$ (5)

Mandelbrot points out that the upper frequency ranks are heavily influenced by *β* due to their low rank values, which should improve the goodness-of-fit of the Equation for the initial segments of frequency-rank distributions [117]. More importantly, Mandelbrot also relates the value of *s* to lexical diversity, holding that the former’s variation is inversely proportional to the latter and can as such “provide a useful measure of vocabulary efficiency with possible applicability to the measurement of intelligence and the detection of certain pathological mental conditions.” [112]

The mere possibility of such practical applications of was however seriously questioned by Gustav Herdan, one of the most vivid critics of the frequency-rank distribution research program. In his argument against the law-like status and scientific validity of both Zipf and Mandelbrot’s formalization attempts [118], Herdan asserts that the main practical defect of both “laws” is that they do not take into account “the possible influence of sample size upon the parameters into account.” [118].

It is simply an empirical fact that the word frequency distribution changes its shape with sample size, which must have the consequence of the parameters changing accordingly. Without taking this into account, comparisons are valid only between samples of the same size from the different languages, or different texts, and quite useless for more general cases. Until the influence of text length (sample size) upon the parameters, and thus the transformation formulae when changing the sample size, are known, these models are of no practical value. [118]

Along with Mandelbrot’s attempts, many other distribution-based models were proposed in the wake of Zipf’s work. Most notable attempts involved among others log-normal distributions [119, 120], generalized inverse Gauss-Poisson models [121], Waring and Generalized Waring distributions [122, 123], as well as a Z-distribution generalization of earlier distribution models [91, 124–129]. In attempting to evaluate whether these indicators “remain constant regardless of the length of the text being analyzed,” [87] Baayen applied them to the word frequency distributions of various texts [88]. The results of these quantitative model comparisons is clear: no single model fully captures the relationship between word types and tokens; instead, “which model is best depends on which corpus is examined.” [116]

We find that the Yule-Simon model is the best choice for Carroll’s *Alice’s Adventures in Wonderland* and Well’s *War of the Worlds*, that the extended Zipf’s law [(Mandelbrot’s canonical form)] outperforms the other models for Carroll’s *Through the Looking-glass*, than the lognormal model is superior for Conan-Doyle’s *Hound of the Baskervilles*, and that the generalized inverse Gauss-Poisson model is preferable for the subcorpus of the British National Corpus [88].

Moreover, Tweedie compared the performance of 12 different lexical diversity indicators over segments of increasing length for 16 distinct corpora, from which they concluded that “the assumption that measures of lexical richness are independent, or roughly independent of text length is invalid.” [130] Thus, no single model has been so far able to fully capture word frequency distributions in their complexity nor diversity.

No simple law can be the full story behind word frequencies because of the complexities of the frequency rank / frequency curve. Therefore, comparisons between simple models will inevitably be between alternatives that are both “wrong” [116].

These considerations certainly put the lawfulness of Zipf’s law in doubt, the latter seeming more akin to a rough empirical tendency than a fully-fledged statistical law [131–133]. However, the fact that “word frequencies… show a statistically reliable structure beyond Zipf’s law that likely will not be captured with any simple model” [116] doesn’t mean that it can’t be modelled. Of particular relevance here is the often-reported misfit of frequent words by existing models, misfit which hints at the statistical specificity of lexical distribution heads and tails. As support for that claim, Ferrer i Cancho [133] found that the frequency-rank distribution of the British National Corpus follows two different exponents, namely *s*_1_ ≈ (−)1 and *s*_2_ ≈ (−)2 for ranks 1 < *N* ∈ (10^3^, 10^4^) and 1 ≥ *N* respectively [133]. While this dual regime system has also been observed elsewhere [134], Tuldava [135] reported that the lemma distribution of A.H. Tammsaare’s novel “Truth and Justice” follows three different regimes: a *s* = (−)0.7 regime for words of frequency rank *O*(*R*) <= 30, a *s* = (−)1.1 regime for lemmas of rank range ]30 − 1,500[, and a *s* = (−)1.4 regime for words of rank *O*(*R*) >= 1,500.

In light of these findings, multiple regime models seem capable of capturing the elusive complexity of lexical distributions. Regarding the measurement of lexical diversity, these considerations are highly significant: while the search for an indicator “that would remain constant regardless of the length of the text being analyzed” [87] is still pending, taking into account the possible multiplicity of distributional regimes might allow for more rigorous and reliable measurements. Given these observations and in a way reminiscent of those Duployan stenographers who proposed specific shorthand subsystems for more frequent words, the present research aims to assess lexical diversity in scientific titles using a new indicator based on the formal distinction between zipfian frequency-rank distribution head and tail regimes.

## Methodology

The python scripts and the aggregated data used for the completion of this research was collected by extracting relevant information of all 31,631,340 articles contained in the Web of Science (WoS) database from 1975 until 2014 inclusively and as of August 31th, 2016. Since the Arts and Humanities Citation Index was created in 1975, we deemed best to start our analysis that year, allowing our comparative assessment of lexical concentration in scholarly articles to span a period of 40 years. All data and scripts used in order to generate the different lexical distributions necessary to that aim can be accessed via the Open Science Framework at osf.io/hxrua.

Discipline assignation was done using the NSF field classification of journals used in the Science and Engineering Indicators (SEI) reports. Since the NSF classification assigns only one discipline to each journal, this prevents the double counting of papers. Articles published in journals of 13 different NSF fields were grouped in 3 disciplinary groups (DG), namely Natural Sciences & Engineering (NSE), Social Sciences & Humanities (SSH) and All Sciences (All). All publications pertaining to Arts were excluded from the study, as their relatively low number prevented any significant longitudinal analysis. A descriptive account of the extracted data is given in Table 1.

**Table 1:** Descriptive statistics of extracted article titles.

	NSF Disciplines	Articles		Journals	Character-Article Ratio	
		Σ	Δ%	Σ	*μ*	Δ%
**NSE**	Biology	2.32e6	417.91	1798	71.47	38.25
	Biomedical Research	3.87e6	373.43	1504	74.56	22.50
	Chemistry	3.31e6	305.77	772	76.45	24.84
	Clinical Medicine	8.66e6	445.73	4299	69.88	35.26
	Earth&Space	1.52e6	655.40	903	64.48	47.62
	Engineering&Technology	3.79e6	646.33	2445	57.01	50.03
	Mathematics	9.24e5	489.53	656	49.80	24.61
	Physics	3.35e6	304.43	623	60.54	23.56
**SSH**	Health	4.57e5	1276.40	610	58.21	54.65
	Humanities	9.00e5	189.46	1799	45.71	21.80
	Professional Fields	8.26e5	276.25	1328	52.09	45.61
	Psychology	6.41e5	289.47	668	60.34	33.98
	Social Sciences	1.07e6	293.64	1691	49.70	40.01
**DG**	NSE	2.77e7	424.51	13000	67.76	31.36
	SSH	3.89e6	305.71	6096	52.12	49.57
	All	3.16e7	401.18	19096	65.58	34.90

Σ = Sum, *μ* = Mean, Δ% = Difference in percentage between 1975 and 2014

Overall, while the annual article count for the whole corpus grows by more than 400% over the [1975, 2014] period, the growth for NSE articles is 38.86% higher than that of SSH articles. Clinical Medicine and Health respectively represent the disciplines with the highest and lowest number of articles extracted from the WoS database, the former having more than 138 times more articles than the latter. Health articles have however experienced the most significant growth, their annual frequency rising by more than 1200% over that period; by contrast, the annual article count for Humanities only increased by 189%. With regard to the different journals involved in the data collection process, Clinical Medicine and Health stand out once again, as the database for this research contains articles from 7 times more journals of the former discipline than of the latter. Finally, article titles in the dataset contain on average a little more than 65 characters. Article titles in NSE generally have longer character strings than in SSH, and the average for the different disciplines and disciplinary groups fall within the ]45, 77[ range, Humanities and Chemistry being on each end of the spectrum. Interestingly, the number of characters by article title extracted has increased by almost 35% during the period observed, which means that article titles have gotten longer during the observed period. The lengthening of SSH titles is rather impressive in that regard, since the Characters-Article Ratio for that disciplinary group almost doubles between 1975 and 2014. At the disciplinary level, increases greater than 50% in Characters-Article Ratio scores for Engineering & Technology as well as Health can be observed; at the other end of the spectrum, Ratio scores for Biomedical Research, Chemistry, Mathematics, Physics, and Humanities are all below 25%. Such generalized lengthening of scholarly article titles does not however imply that the number of word tokens by article also grows during the observed period; before this claim can be validated, various linguistic processing operations are required.

In order to convert character string into lists of words, all titles were tokenized using the Treebank tokenizer [136, 137] of the Natural Language ToolKit *nltk* [138]. Following conversion to lower case, all words were split at dashes, colons, and slashes, while leading and trailing spaces, digits as well as punctuation symbols were removed. Finally, French, German, Spanish and Italian stop words were removed from the lexicon.

In order to allow for preliminary analyses, annual token counts for each discipline and disciplinary group were compiled in other to assess the evolution of the number of tokens by article as well as the number of tokens for each word type over the years. The results of these various data collection and preprocessing operations are presented in Table 2.

**Table 2:** Descriptive statistics of article titles corpus, disciplinary group sub-corpora and disciplinary sub-corpora.

	NSF Disciplines	Tokens-Article Ratio		Type-Token Ratio	
		*μ*	Δ%	*μ*	Δ%
NSE	Biology	9.47	36.28	0.11	-50.42
	Biomedical Research	9.73	26.36	0.07	-42.89
	Chemistry	9.98	26.07	0.077	-48.67
	Clinical Medicine	9.00	40.11	0.04	-58.32
	Earth&Space	8.82	46.64	0.10	-58.55
	Engineering&Technology	7.75	48.03	0.06	-63.85
	Mathematics	6.51	32.86	0.09	-53.20
	Physics	8.29	26.36	0.05	-39.26
**SSH**	Health	7.76	68.64	0.16	-78.25
	Humanities	6.52	22.78	0.22	-25.02
	Professional Fields	6.86	48.60	0.12	-51.85
	Psychology	7.74	39.46	0.13	-49.81
	Social Sciences	6.67	46.20	0.13	-47.47
**DG**	NSE	8.93	33.44	0.04	-55.53
	SSH	7.00	47.41	0.098	-57.85
	All	8.66	36.36	0.04	-56.91

Σ = Sum, *μ* = Mean, Δ% = Difference in percentage between 1975 and 2014

Tokens-Article Ratio scores show that, over the [1975, 2014] period, the number of word tokens by article title for the different disciplines and disciplinary groups all fall within the ]6.50, 10[ range. NSE article titles are generally wordier than their SSH counterparts, and Mathematics and Chemistry have respectively the lowest and highest tokens-article ratio. In the case of Characters-Article Ratio, the minimum score was obtained by Humanities, which means that Mathematics tend to have shorter title words than the former discipline. More importantly, the average annual word token count for the article titles collected increase by 36% over the observed period, which means that article entitling has gone wordier over the years. This trend is very telling as regards to the annual TTR scores obtained for the different disciplines and disciplinary groups. At first sight, the mean annual TTR values calculated for each disciplinary group and discipline sub-corpus show an interesting variation in concentration. At the global level, the title vocabulary of SSH articles appears more diversified than NSE articles, as they include two and a half times more word types for the same amount of word tokens. At the disciplinary level, an even sharper contrast can be observed for Clinical Medicine and Humanities, whose titles respectively have the highest and lowest concentration. However, the universal decrease in TTR scores over the whole period put into question the reliability of the scores obtained as well as the significance of any interpretation thereof. Indeed, the fact that this generalized plummeting of TTR scores coincides with a universal increase in corpus size immediately calls to mind the above-mentioned and empirically-validated sample size dependency issues. Worse still and according to R2 analyzes, variation in annual word token counts for the majority of different disciplinary group and disciplinary sub-corpora can explain more than 90% of the variation in corresponding TTR values. In light of this, no definitive conclusion regarding lexical diversity in scientific titles can be reached without first controlling for corpus size dependency. This objective represents the main focus of this methodological section, which first describes the formal specifications of our new dual-regime lexical diversity indicator, followed by a comparative assessment of the corpus size dependency of head and tail regimes on random samples of varying sizes based on the 2014 WoS title word sub-corpus, and then on the full Wos article titles corpus.

### Development of a dual-regime zipfian frequency-rank lexical diversity indicator

**Fig 1:**
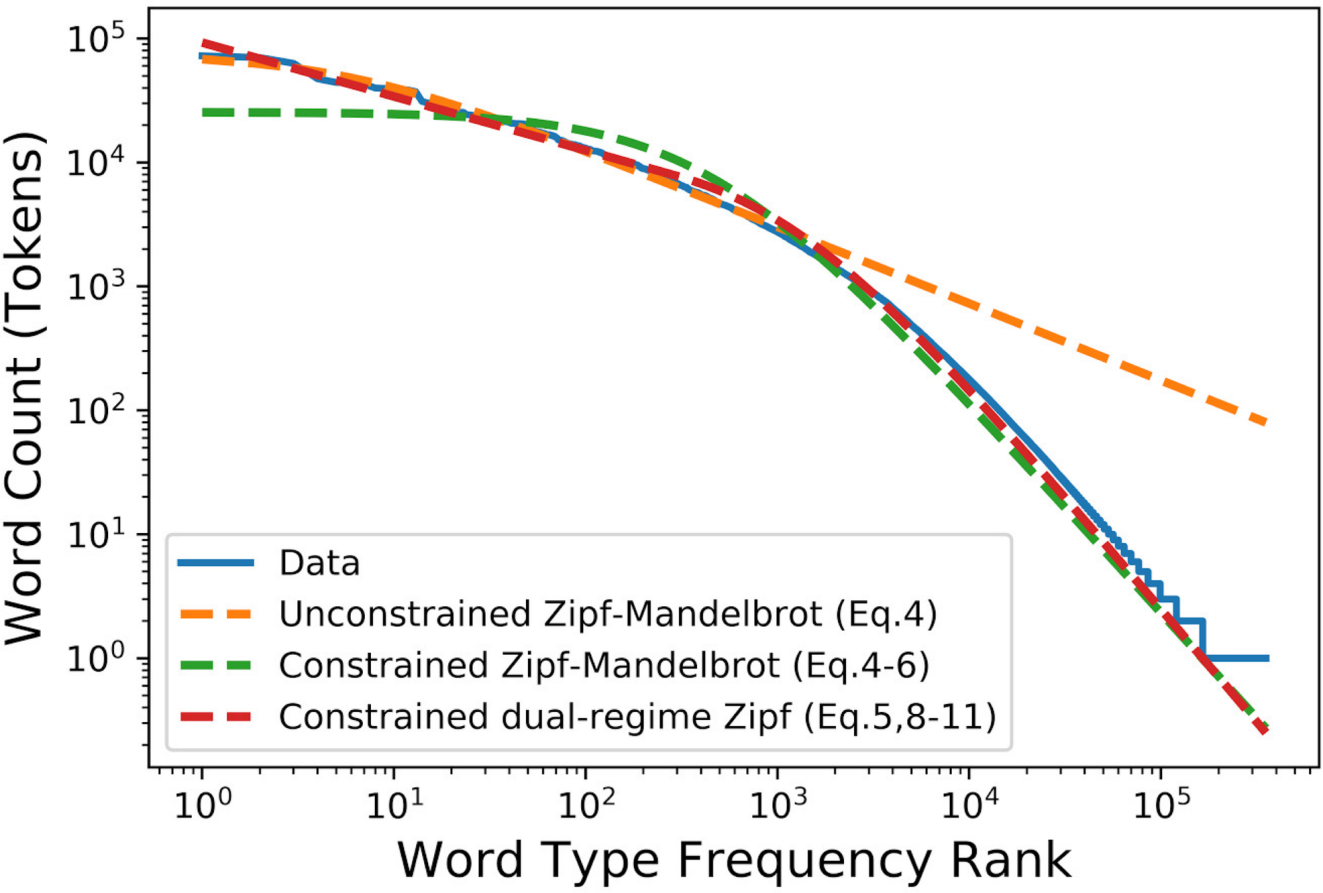
Various fits for the frequency-rank distribution of 2014 WoS title words.

The distribution tail-based indicator created and used in this project is the result of a stepwise process: various zipf-based fits were developed and tested on the 2014 WoS title words corpus, which contains a total of 15,255,299 word tokens distributed over 352,958 word types. Results of these tests are shown in Fig 1.

We first start by calculating the value of *s* from Mandelbrot’s canonical law (Eq (5)). As shown by the unconstrained single-regime fit in Fig 1, this law does a poor job at fitting the distribution: while it seems adequate for the first hundreds of the most frequent word types, as Mandelbrot intended, the same cannot however be said of the distribution tail, which contains by far the largest number of both word types and tokens.

A way to correct for this is to fit the parameters of the canonical law with two constraints: the word type count *n* and the word token count *N*. Those two constraints are mathematically and respectively expressed by Eqs (6) and (7).

$$\begin{array}{c} O\left(R=n\right)=\frac{c}{{\left(n+\beta \right)}^{s}}=1 \end{array}$$ (6)

$$\begin{array}{c} \int_{0}^{n}O\left(R\right)dR={\frac{c{\left(R+\beta \right)}^{\left(1-s\right)}}{\left(1-s\right)}|}_{R=0}^{R=n}=N \end{array}$$ (7)

As shown by the constrained single-regime fit in Fig 1, these constrains ensure an adequate tail fit, but the model tends to oscillate around the distribution head. In our opinion, this wobbling come from the fact that we are trying to apply a single-regime fit to the whole corpus, whereas the slope on Fig 1 is not constant according to the rank of the word. As suggested earlier, a possible solution to this problem would be to compute and join two distinct regimes, one for the distribution head and another for the tail. Such dual-regime model would be represented by a function that explicitly allows different exponents depending on the rank of the word. The simplest function that allows this behaviour would be to allow for two different zipfian regimes, separated around rank *l*, as illustrated in Eq (8). Here, Θ is the Heaviside step function where Θ(*x*) = 0 if *x* < 0 and Θ(*x*) = 1 if *x* > 0:

$$\begin{array}{c} O\left(R\right)=\frac{\Theta (l-R)c}{{\left(R\right)}^{s}}+\frac{\Theta (R-l)d}{{\left(R\right)}^{t}} \end{array}$$ (8)

However, this model’s derivative is discontinuous as point *R* = *l*. Therefore, one of those regimes should obey the canonical law to allow the continuity of the derivative. Therefore, the regime where *R* > *l*, called the Zipf tail regime, will obey Mandelbrot’s canonical law, and the regime where *R* < *l*, referred to as the Zipf head regime, will obey the simpler but exponentially different Zipf law.

This choice can be justified by the fact that since there normally is a small number of distinct frequent words, it would therefore be very easy for a single point to affect the fitted model, thus jeopardizing the indicator’s stability. Also, if both regimes obeyed the Zipf-Mandelbrot law, we would be at a higher risk of having too many free parameters. The model is shown in Eq (9) where *c*, *d*, *r*, *s*, *t*, and *l* are parameters to be fitted (for more information on the fitting of Eq (9), see S1 Appendix in the Supporting Information section).

$$\begin{array}{c} O\left(R\right)=\frac{\Theta (l-R)c}{R^{s}}+\frac{\Theta (R-l)d}{{\left(R+r\right)}^{t}} \end{array}$$ (9)

While the constraint of Eq (6) still holds, Eq (7) must be rewritten as

$$\begin{array}{c} \int_{0}^{n}O\left(R\right)dR={\frac{cR^{1-s}}{1-s}|}_{R=0}^{R=l}+{\frac{d{\left(R+r\right)}^{1-t}}{1-t}|}_{R=l}^{R=n}=N \end{array}$$ (10)

On top of those, two other constraints are also added to this model due to continuity. Indeed, the model should be continuous, and have a continuous derivative at the junction of the two regimes where *R* = *l*. The continuity and derivative continuity are shown in Eqs (11) and (12), respectively.

$$\begin{array}{c} \frac{c}{l^{s}}=\frac{d}{{\left(l+r\right)}^{t}} \end{array}$$ (11)

$$\begin{array}{ccc} \frac{cs}{l^{s+1}} & = & \frac{dt}{{\left(l+r\right)}^{t+1}}\\ \frac{s}{l} & = & \frac{t}{l+r} \end{array}$$ (12)

One can note that there are six parameters in Eq (9), but four constraints in Eqs (6), (10), (11) and (12). Therefore, our fit has only two free parameters, which is appropriate for a model with two regimes. There however is no analytical solution for this set of Equations, which means that the constrained fit must be done numerically. As shown by the constrained double regime Zipf fit in Fig 1, the use of this dual-regime fit offers by far the best results on the test corpus.

### Corpus size dependency evaluation

**Fig 2:**
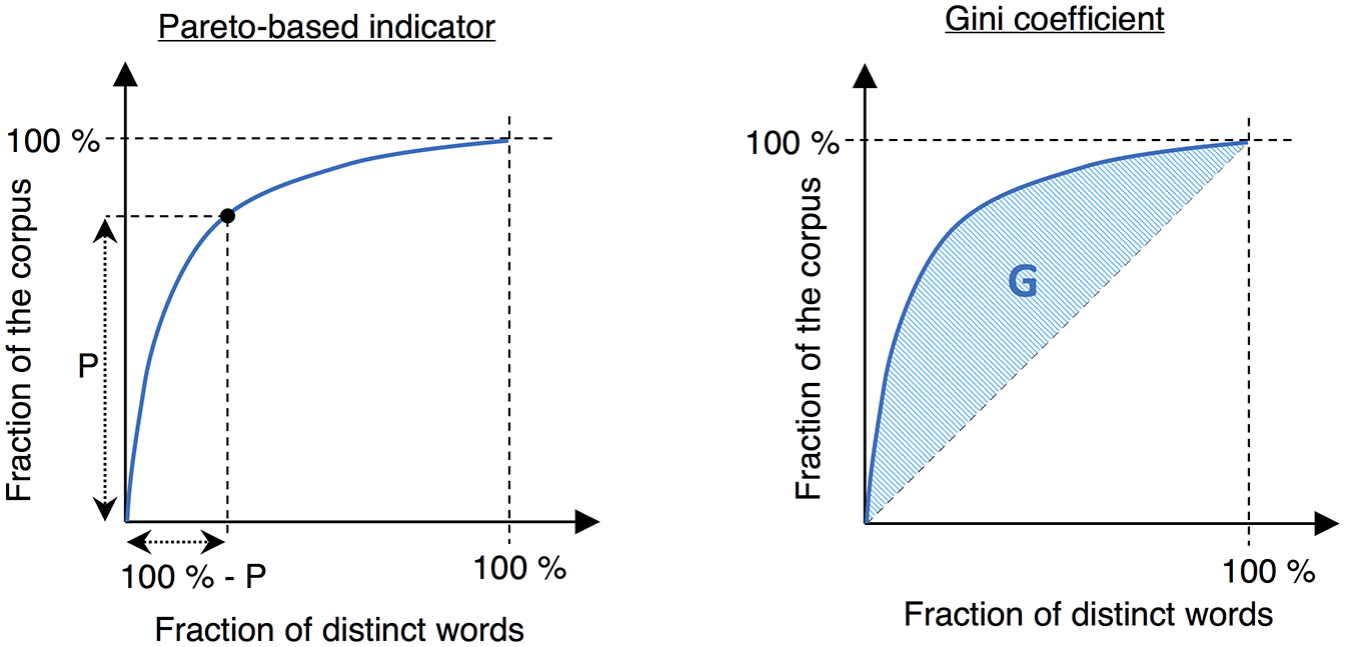
Graphical representation of the Pareto-based indicator and the Gini coefficient on cumulative distributions.

In order to evaluate the performance of both zipfian distribution head and tail fits as regards to corpus size dependency, comparisons with existing measures on cumulative distributions are here carried out. Three different indicators were implemented and tested, along with the new indicator. The first and most obvious one is the TTR, which is rather easy to understand and is still used today as an indicator of lexical diversity. The two others are often used in economics as resource concentration indicators: the Pareto law and the Gini coefficient. Their graphical representation is shown in Fig 2.

The Pareto law, also known as the 80/20 rule or the principle of factor sparsity, states that “a small number of causes (20%) is responsible for a large percentage (80%) of the effect.” [139] This principle is of obvious relevance to lexical distributions, as a few frequent word types account for a large proportion of word tokens. Based on this principle, a concentration indicator can be developed by taking the value *P* of a curve where (100% − *P*) of the word types account for *P*% of all word tokens. The higher *P* is, the more concentrated the distribution is. This indicator is illustrated on Fig 2.

**Fig 3:**
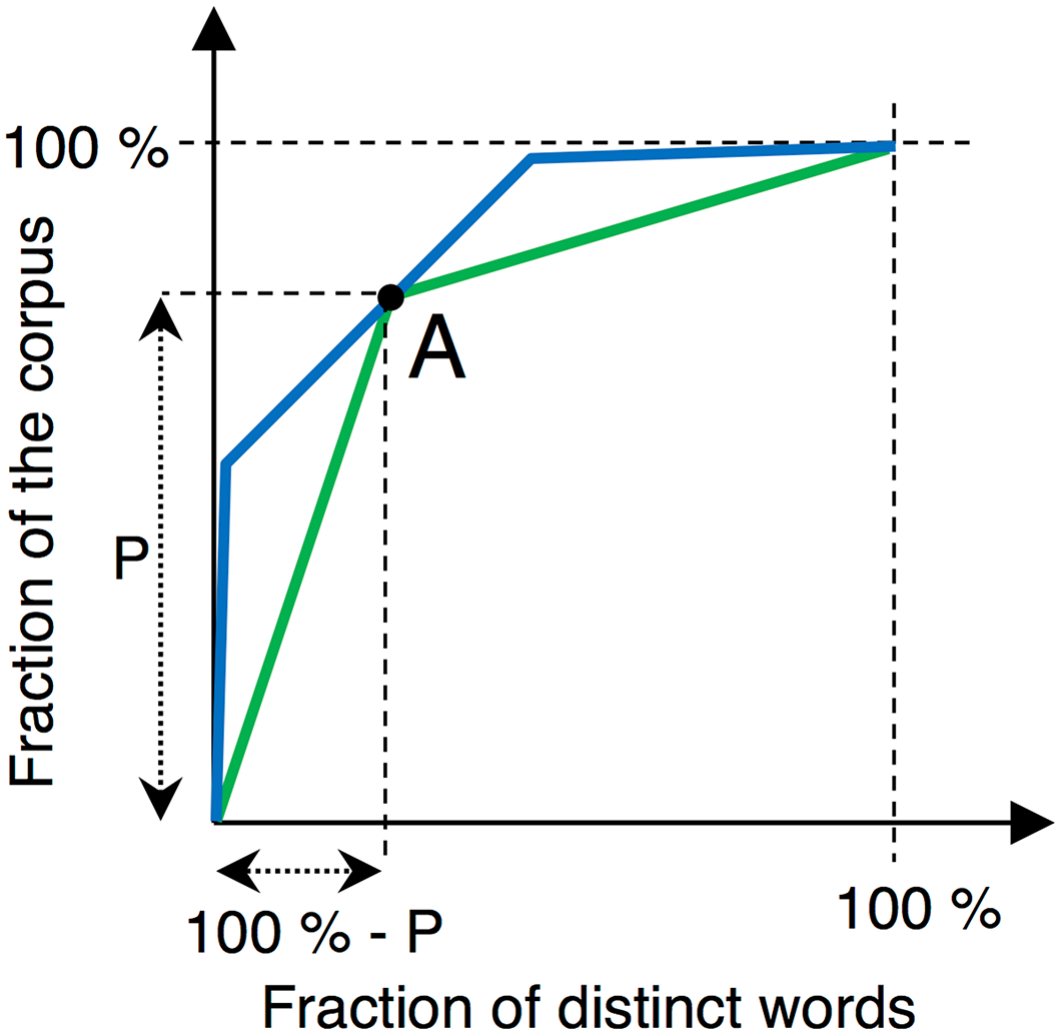
Distribution of two different corpora with identical Pareto-based indicator value.

However, this *P* concentration value only considers a single point on the distribution curve and cannot therefore account for effects on others points. As seen on Fig 3, if a Pareto-based indicator is calculated at point A, it cannot differentiate between the two plotted curves that are clearly different. The Gini coefficient solves this issue by considering the area below the curve, as shown in Fig 2. Normalized between 0 and 1, the Gini coefficient indicates a high concentration of the distribution if close to 1.

Both the Pareto-based and the Gini indicators are strangers to the field of statistical linguistics. However, the evaluation of their sample size dependency, together with that of the TTR and the dual-regime indicator, might allow for interesting and unprecedented comparisons and insights.

As regards to the evaluation procedure, finding two corpora of different size but with the exact same lexical diversity for testing purposes is next to impossible. A more feasible option consists in extracting a random sample of any size from the analyzed corpus, and check whether or not the lexical diversity indicator is affected by this procedure. Since the diversity of a corpus’ lexicon should not change whether calculated on the whole corpus or on any random subcorpus, the sample size dependency of indicators can be evaluated on the basis of their stability in this regard.

**Fig 4:**
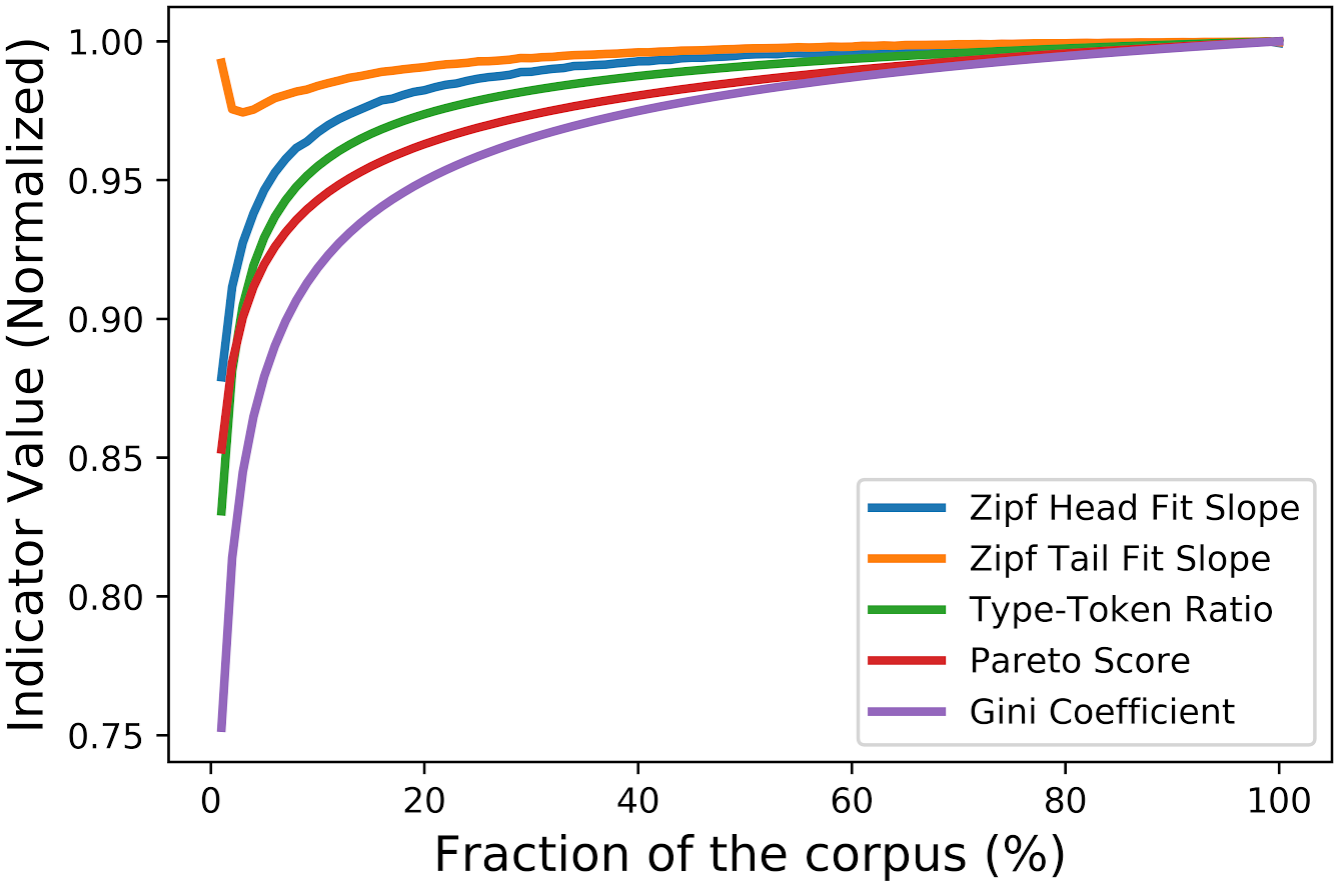
Pareto-based, Gini, TTR, Zipf head and tail scores of random corpus samples of increasing size. All parameters were normalized to 1 for the full corpus.

Using the same test corpus, 100 random samples of size *N* ∗ *X*% were extracted for each percentage value *X* between 1% and 100%. TTR, Pareto-based, Gini, and dual-Zipf scores of each random sample were then calculated and averaged over same-size samples. The results of these tests are shown on Fig 4 and Table 3. In the case of the Pareto-based, Gini, and TTR indicators, the concentration clearly drops as sample size gets bigger. The Pareto-based indicator drops by 1.4% and 5,7% for samples respectively half and a tenth of the size of the corpus. For the Gini coefficient, those drops are even greater, corresponding to 1.8% for a sample half the size, and to 8.2% for a sample a tenth of the size. Despite its simplicity, the TTR surprisingly has the smallest drops of the three: 0,9% for a sample half the size, and 4,5% for a sample a tenth of the size.

**Table 3:** Value drops of various lexical diversity indicators for different sample sizes.

Sample size	Gini	Pareto	Type-Token	Zipf-frequent	Zipf-rare
50%	1,8%	1,4%	0,9%	0,4%	0,2%
10%	8,2%	5,7%	4,5%	3,2%	1,5%

The corpus size dependency of these indicators can become highly problematic in longitudinal studies, as annual sub-corpora can drastically vary in size over time. Given the fact that in the present corpus, the annual number of word tokens grows by 547% over the [1975, 2014] period, one can only presume that the performance of the Gini, Pareto, and TTR indicators should be greatly affected by annual variations in subcorpus size.

By contrast, the head and tail regimes are a lot more stable than the TTR, Pareto-based and Gini indicators. Indeed, if we take a sample that is a tenth of the size, the head and tail regime scores respectively drop by 3,2% and 1,5%, while their value only drops by 0,4% and 0,2% for samples half the size of the test corpus. While total sample size dependency has not yet been achieved, this performance of exponents *s* and *t* from Eq (9) is still impressive and to our knowledge benchmark-setting. In our opinion, this unprecedented size independency of head and tail regime fits warrants their use as lexical diversity measures in general, but also in assessing the evolution of lexical concentration in frequent and rare words regimes in the longitudinal corpus under study.

### A comparative assessment of lexical diversity indicators on the full WoS title words corpus

In order to analyze the evolution of lexical diversity in the different WoS disciplines, the values of the different indicators presented in the previous section were calculated for each year between 1975 and 2014 inclusively. In the case of Health and Mathematics, annual word type and token counts before 1995 and 1981 were respectively excluded because of their relatively low frequency. Indeed, in cases where the annual token count is below 65,000, the number of different word types that occur only once or a few times is enormous, resulting in distribution plateaus which make zipfian fits behave erratically and randomly manner, making it thus extremely difficult to distinguish between head and tail regimes, if regimes there are (for an illustration of this phenomena, see S1 Appendix in the Supporting Information section).

**Fig 5:**
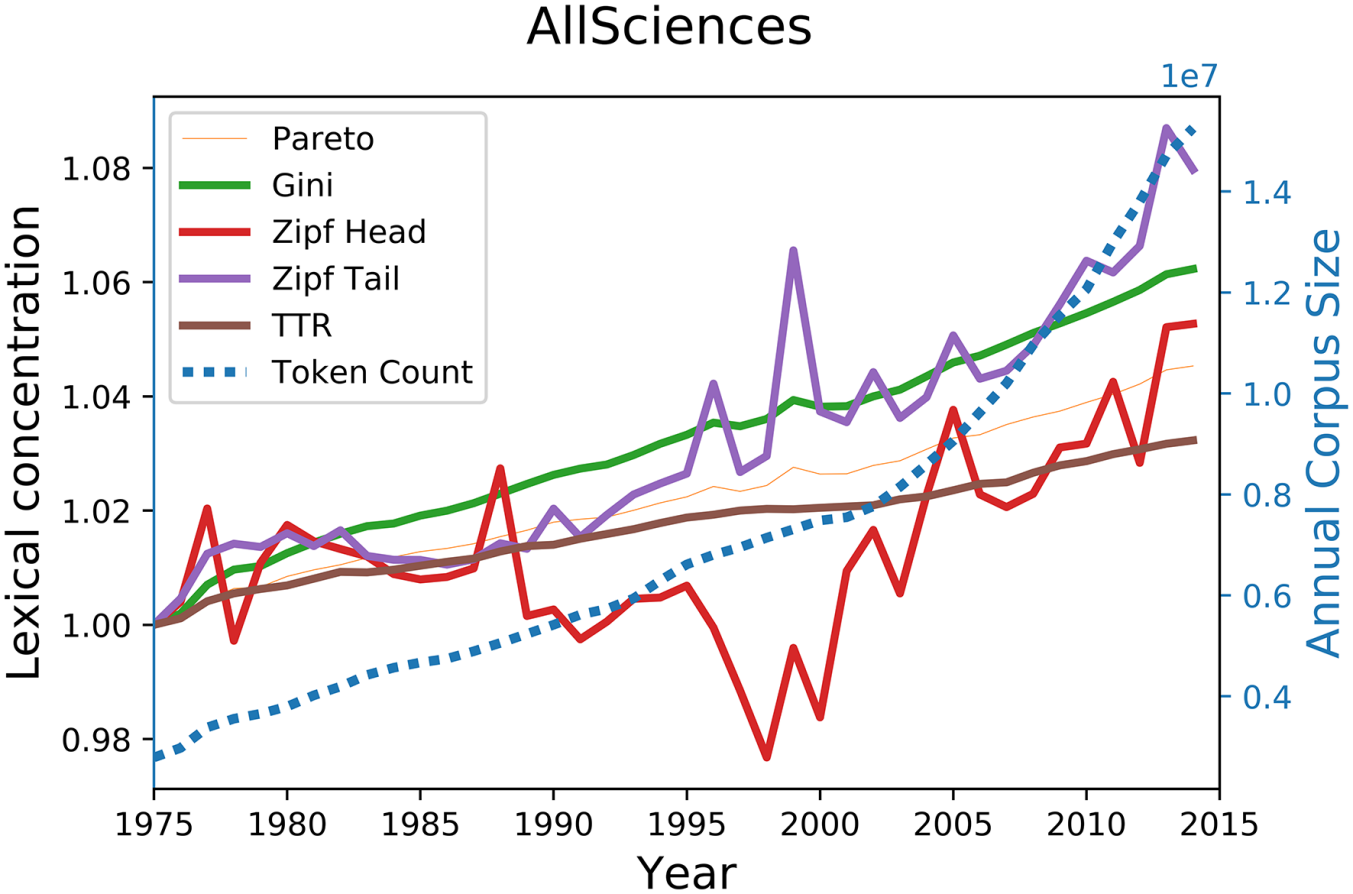
Evolution of lexical diversity indicator scores on the WoS title words corpus. The evolution of the size of the corpus is shown in blue. All parameters were normalized to 1 for the 1975 corpus.

Results of these computations on the whole corpus are shown on Fig 5; results for NSE, SSH, and their affiliated disciplines are respectively shown in S2 Appendix, S3 Appendix, and S4 Appendix in the Supporting Information section. What is most striking at first glance is how radically different the behaviour of both zipf head and tail regimes are compared to the other indicators used in this study: while the Pareto-based indicator, the Gini coefficient, and the TTR maintains a overall steady rise between 1975 and 2014, both Zipfian indicators fluctuate greatly over the same period, showing spikes and sudden drops when other indicators remain adamant in their slow rise. Moreover, the score of traditional indicators’ all show slight twitches in 1996 and 1999, but such flutterings are hard to make out and are drowned in the general upward slope of these indicators; in contrast, the rare words exponent show the same perturbations much more clearly, while also showing fluctuations completely hidden with the previous indicators, such as the spikes observed for 2002 and 2005.

**Fig 6:**
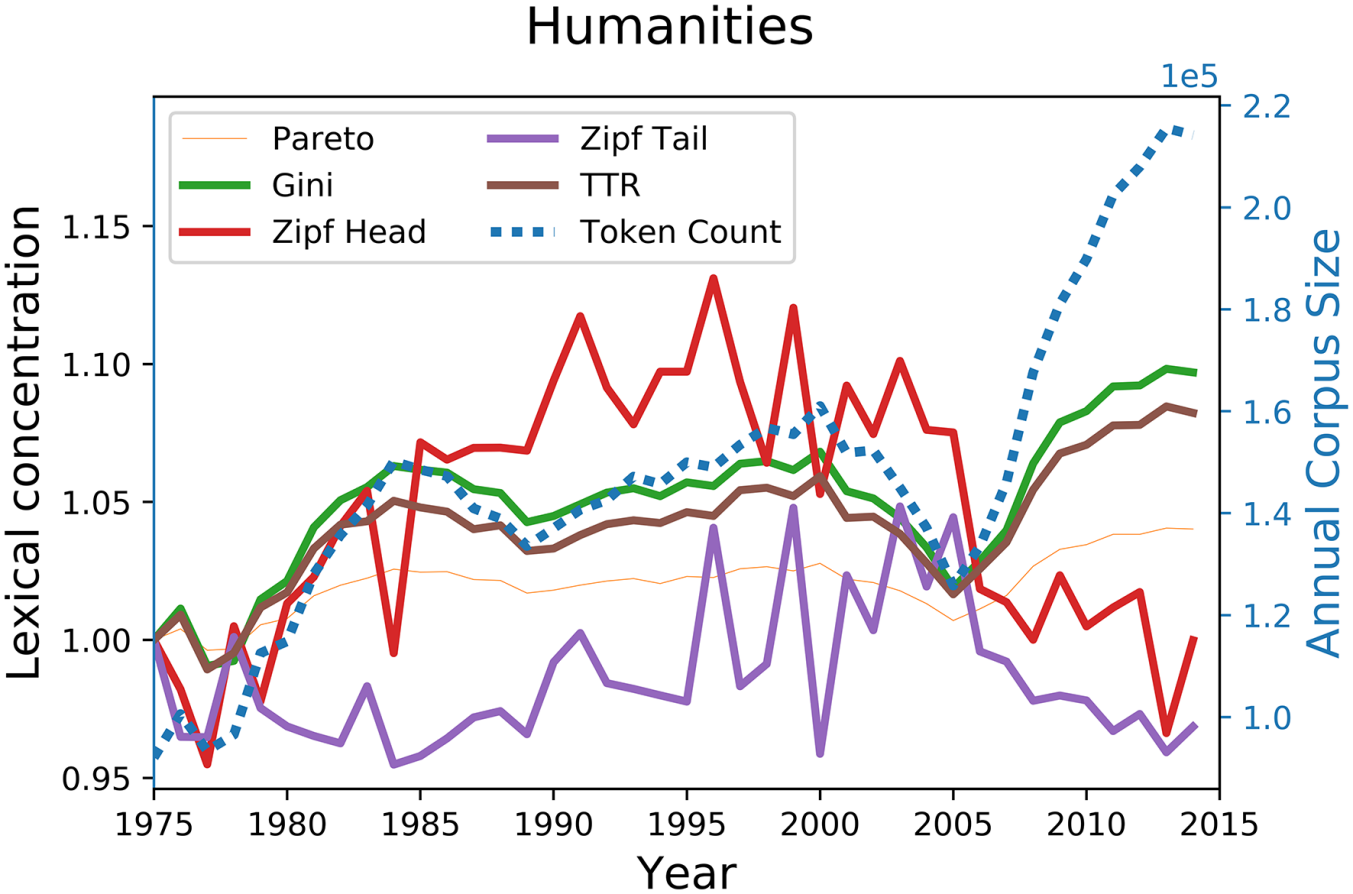
Evolution of lexical diversity indicator scores on the WoS title words sub-corpus for Humanities. The evolution of the size of the corpus is shown in blue. All parameters are normalized to 1 for the 1975 corpus.

The sharpest difference in behaviour between these indicators is however given by Fig 6, which shows the lexical concentration scores for Humanities article titles. For the period 2005-2013, during which the number of humanities papers almost doubles, the score of previous concentration indicators all increase at different paces, while both head and tail regimes decline over the same period. Similar tendencies can also be observed for Earth and Space as well as for Mathematics between 2000 and 2014.

These different contrasts acquire a special significance in light of the the corpus size dependency of traditional indicators shown in Fig 4. Indeed, since the size of the corpus almost tripled during the observed period, one cannot but conclude that what distinguishes the performance of traditional indicators from that of the zipfian regime exponents developed here is nothing but artificial statistical noise due to corpus size variation effects, which makes them inappropriate for lexical concentration evaluation purposes.

Comparisons with traditional indicators apart, an equally striking difference in behaviour can also be observed for Zipf head and tail regimes. To take but one example from Fig 5, the 1995-2000 period sees head and tail regimes drifting apart, frequent words becoming more diversified as the rest of the distribution increases in lexical concentration. While both regimes offer interesting insights, the tail regime appears better suited for the measurement of lexical concentration. Indeed, frequent words forming the Zipf head regime represent a very small portion of the whole distribution, which means that annual variations of a given frequent word type can easily affect the performance of the zipf head regime. For example, the head regime of the 2014 corpus used in the previous tests accounts for 26.3% of all word tokens, but only 292 (0.08%) of all 352,958 word types, which means that any rise or drop in token count for one of these words in 2015 has the potential of substantially affecting the frequency-rank distribution head regime for that year. Given this and the fact that distribution tail regimes perform best in terms of corpus size dependency, focusing solely on this indicator constitutes the most robust, sensitive, and thus reliable way to measure lexical diversity in scientific titles.

It might here be objected that this strategy leaves a substantial amount of word tokens out of the Equation. For example, in the above-mentioned case of the 2014 test corpus, advocating such an approach means that all frequent word types, amounting up to 26.3% of all word tokens, are not accounted for. However, given that vocabulary range is all about type counts, that distribution heads account for the near totality of word types (99.92% for 2014), and that the only other indicators that perform as well as the zipf head regime as regards to sample size independency are based on incomparably smaller fractions of corpora, “beheading” zipfian frequency-rank distributions for lexical diversity measurement purposes seems like a rather small price to pay for the insights and analyses that approach may provide.

## Assessing lexical diversity in WoS article titles

**Fig 7:**
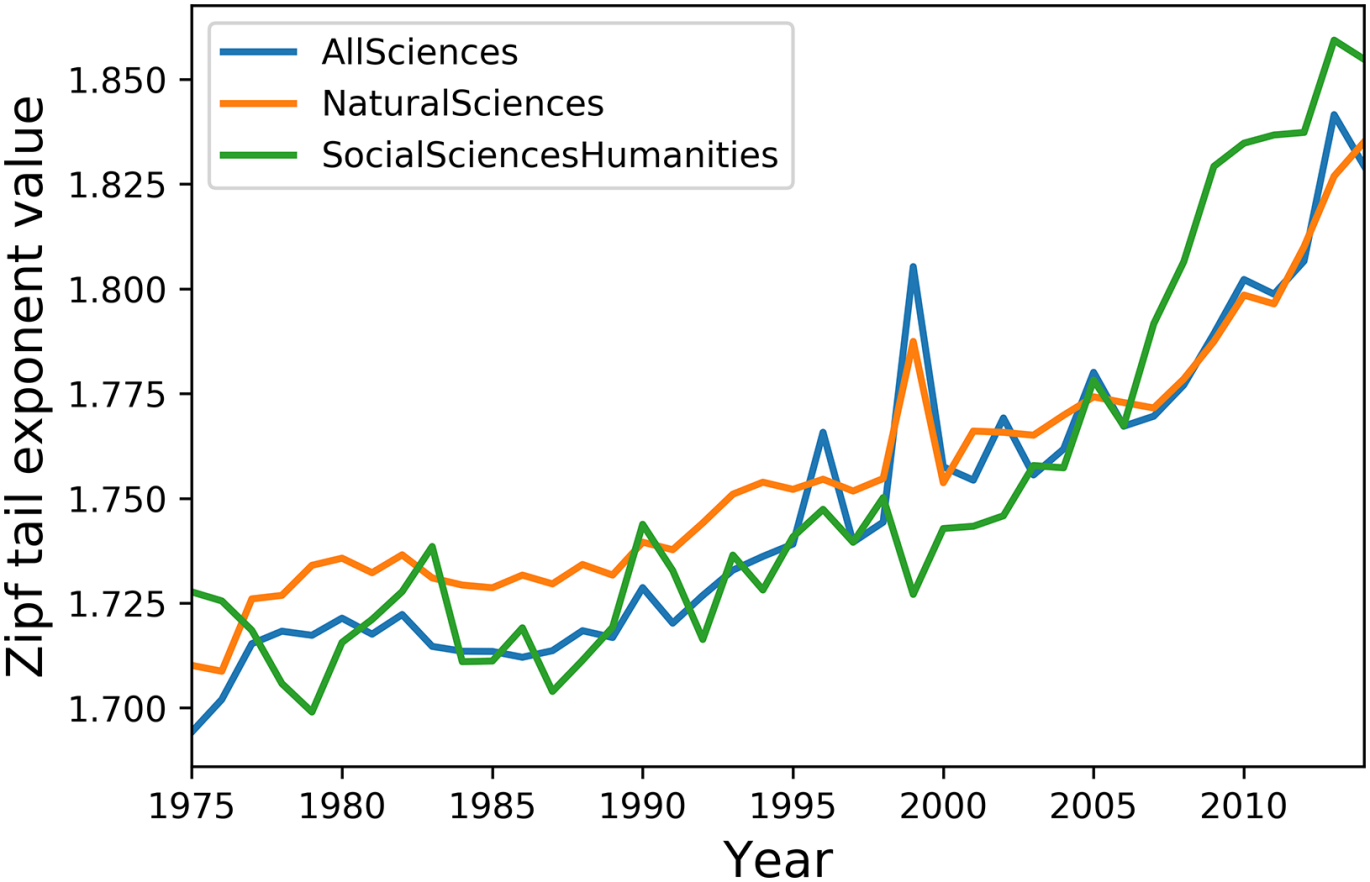
Evolution of the lexical concentration of scientific titles in all Sciences.

As shown by the above tests, the distribution tail fit slope values present the best resiliency to corpus size variation, resiliency which warrants its use in measuring the evolution of lexical concentration or diversity in WoS article titles. Results for the whole corpus as well as for the NSE and SSH sub-corpora are shown in Fig 7.

Overall, the lexical concentration of scientific article title wording increases by 7,97% over the [1975, 2014] period, while the concentration level for NSE and SSH titles are very similar, with respective increases of 7.31% and 7.36%. Such similarity however masks the fact that both disciplinary groups do not have the same leverage over global concentration levels. Indeed, there is good reason to believe that fluctuations in concentration level for all sciences is mainly driven by that of natural sciences: first, NSE title words provide more than 89% of the analyzed corpus, with 285,249,540 out of 319,560,799 words; second and more importantly, concentration level fluctuations in all sciences correlate at *R* = 0.98 with those of Natural Sciences, which is higher than the *R* = 0.88 observed between all sciences and Social Sciences & Humanities. Most importantly, however, fluctuations in both NSE and SSH sub-corpora correlate to each other at *R* = 0.89, 8, and this proximity in lexical diversity evolution between both disciplinary groups seems to hint at the unity and universality of the process of scientific entitling as a whole. However, before closing on this matter, a more fine-grained analysis, focusing on disciplinary patterns from both Natural Sciences and Social Sciences, seems in order.

### NSE disciplines

**Fig 8:**
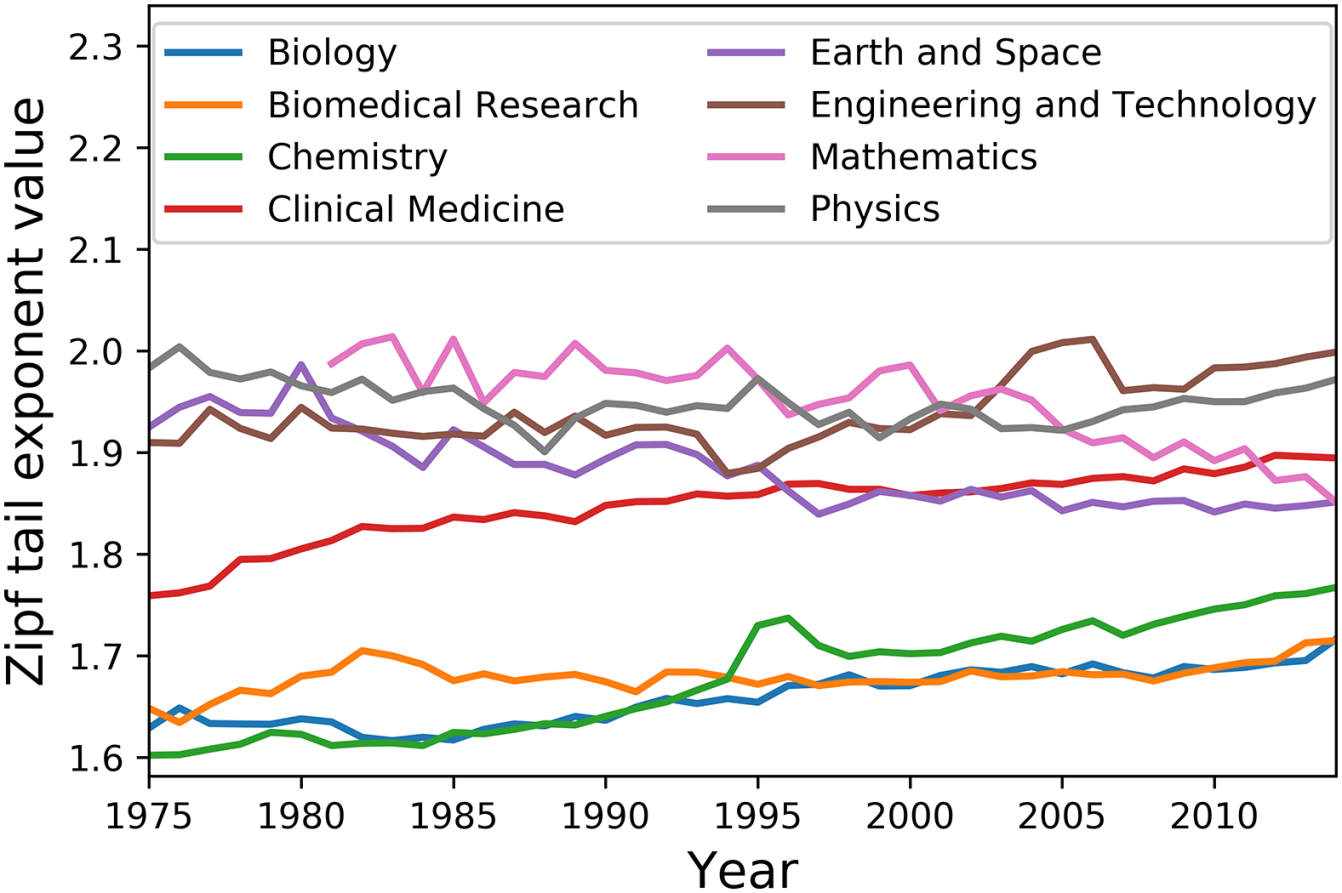
Evolution of the lexical concentration of scientific titles in Natural Sciences and Engineering disciplines.

Concentration values for NSE disciplines are shown in Fig 8, while summary descriptive statistics for each discipline are given by Table 4. At first glance and despite the fact that lexical concentration plotting for Mathematics only begins in 1981, distribution tail regimes maintain relatively steady trends over the [1975, 2014] period. This is also supported by the fact that relative standard deviation scores represent a very small proportion of disciplinary averages.

**Table 4:** Various statistics on the lexical concentration score evolution of the different NSE disciplines.

Discipline	Diff%	*μ*	*σ*	RSD%
Biology	+5,38	1.66	0.03	1.64
Biomedical Research	+4.05	1.68	0.02	0.90
Chemistry	+10.31	1.68	0.05	3.27
Clinical Medicine	+7.71	1.85	0.04	1.91
Earth and Space	-3.83	1.88	0.04	2.00
Engineering and Technology	+4.66	1.94	0.03	1.74
Mathematics	-7.42	1.96	0.04	2.17
Physics	-0.57	1.95	0.02	1.07

Diff% = Difference in percentage over time, *μ* = average, *σ* = standard deviation, RSD% = relative standard deviation

Our lexical concentration indicator also shows that a majority of NSE disciplines increase in lexical concentration over that period. While Chemistry and Clinical Medicine are the most obvious and extreme cases, lexical concentration increases in Biology, Biomedical Research, and Engineering and Technology are also noteworthy. Given that these 5 disciplines account for as much as 80.93% and 72.33% of all title word tokens for the NSE sub-corpus and the whole corpus respectively, one has to conclude that the former disciplines are those most responsible for the lexical concentration increases observed for the two disciplinary groups.

In contrast, a diversification in title wording can be observed in Mathematics, Earth & Space and to a lesser extent Physics. What is most interesting by this trend is that these disciplines are not only the most ancient and emblematic ones, but also those most strongly associated with fundamental research. While the methodological apparatus adopted in the present research prevents any detailed analysis, it is reasonable to assume that the flattening of the distribution tail fit slope in the case of Earth & Space is partly the result of that tail getting heavier due to the discovery and naming of newly discovered heavenly bodies such as constellations, stars or exoplanets. In the case of Mathematics and Physics, it may be hypothesized that the lexical diversification can partially be explained by the prevalence of eponymous nomenclature in these disciplines, as major breakthroughs therein often tend to be named after their discoverer. A closer and comparative look at these disciplinary practices and their impact on research cultures however lies outside the scope of this study.

Overall, these increases and decreases in lexical concentration in all NSE disciplines over the [1975, 2014] period form a two-regime system.

1. On the lower end, Biology, Biomedical Research, Chemistry, Clinical Medicine, and Engineering & Technology titles start at a lower lexical concentration level than those of other disciplines, but increase in concentration over time; Engineering & Technology even becomes the discipline with the highest lexical concentration level in title wording at the end of the observed period.
2. At the upper end, Physics, Mathematics, and Earth & Space article titles start at a higher concentration level, but steadily decrease in concentration as years go by.

Given the steadiness of both trends, the near future may possibly witness a global convergence of lexical concentration of article title wording for NSE disciplines; further research would however be required to validate this conjecture.

### SSH disciplines

**Fig 9:**
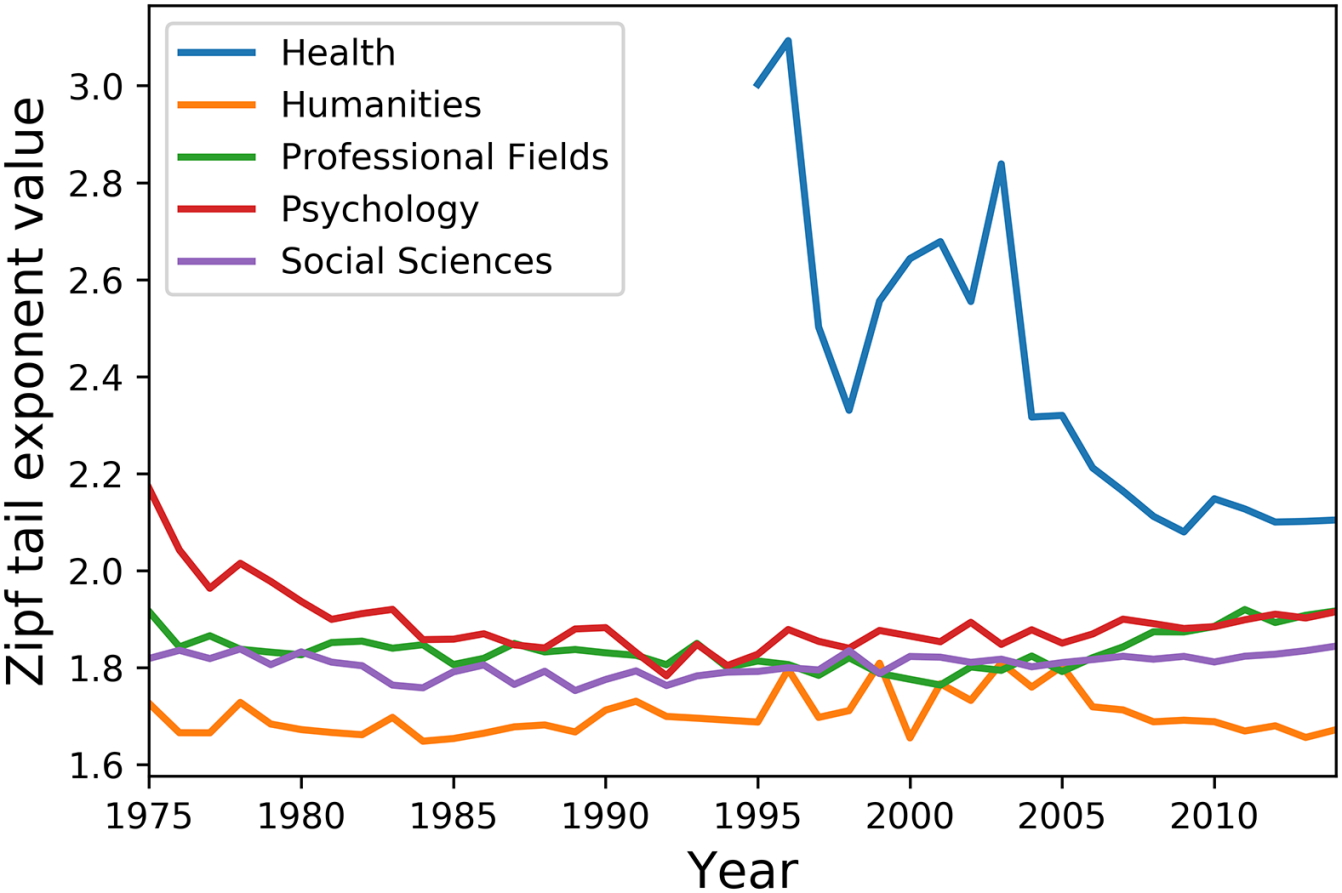
Evolution of the lexical concentration of scientific titles in Social Sciences and Humanities disciplines.

Fig 9 and Table 5 present results for the different SSH disciplines. A quick glimpse at these results show that the evolution of lexical concentration in Health article titles is markedly different from that of all other scientific disciplines. First, plotting only begins in 1995, as the token count for that discipline’s article title corpus was below the fixed threshold before that year. Additionally, the variance in lexical diversity observed for that discipline is greater that that of all other disciplines combined, including those from NSE. However, given that such fluctuations start to dampen from 2005 onward, the strong oscillations observed in the first decade plotted could simply reflect a data collection artefact created by the haphazard or topic-based inclusion of journal data in the WoS database. Nonetheless, following this initial haywire period and up to 2014, the lexical concentration level in health article titles is still markedly higher than that of other disciplines. This could be explained by the fact that publications classified as health journals in the WoS database form a topically coherent, health-focused, and thus concentrated lexicon compared to other disciplinary corpora, which contain specialities with widely diverging terminologies and discourses, for example Management and Education in Professional Fields or Tropical Medicine and Arthritis in Clinical Medicine.

**Table 5:** Various statistics on the lexical concentration score evolution of the different SSH disciplines.

Discipline	Diff%	*μ*	*σ*	RSD%
Health (1995 onward)	-29.92	2.4	0.32	13.28
Humanities	-3.14	1.70	0.04	2.60
Professional Fields	0.06	1.84	0.04	2.13
Psychology	-11.73	1.89	0.07	3.59
Social Sciences	+1.40	1.81	0.02	1.31

Diff% = Difference in percentage over time, *μ* = average, *σ* = standard deviation, RSD% = relative standard deviation

Turning to other SSH disciplines, despite the fact that Humanities article titles have a slightly more diversified vocabulary, evolution of lexical concentration in the former’s lexical concentration is remarkably uniform and stable over the [1975, 2014] period. Indeed, the mean concentration values for Humanities, Professional Fields, Psychology, and Social Sciences over time is confined within the [1.70, 1.90[ range, while the standard deviation values never exceed 3.6% of the recorded mean for any one of these disciplines. While such steadiness differs from the trends observed for the different natural sciences disciplines, it also sharply contrasts with the 7.36% increase in lexical concentration observed for the whole SSH corpus and shown in Fig 7. Indeed, while the equivalent increase in lexical concentration for the Natural Sciences corpus can be explained by the title wording evolution of its biggest constitutive disciplines, no such explanation can be given for SSH disciplines: only the Professional Fields and Social Sciences sub-corpora positively correlate with the SSH sub-corpus in terms of lexical concentration evolution, and their respective scores are only *R* = 0.56 and 0.49. Additionally, given the impressive performance of zipfian tail slope fits regarding corpus size dependency issues, variations in corpus size have to be ruled out as explanatory factors. A more likely hypothesis is that the contrast in lexical concentration between the whole SSH corpus and each SSH discipline sub-corpus results from aggregation effects. For example, if word types A and B occur respectively 4 and 2 times in a corpus, while word types A and C occur respectively 5 and 3 times in another, aggregating both corpora increase the relative frequency of word type A and decrease that of word types B and C, resulting in a more asymmetric and thus concentrated distribution. Were such aggregation effect to be a factor in the present case, it would mean that the increase in lexical concentration level observed for the whole SSH corpus over the [1975, 2014] period results in a gradual cross-disciplinary homogenization of the highest occurring word types in the word distribution tails of all SSH disciplines.

Whatever the reason for such whole-part discrepancy is, the fact remains that the lexical concentration in both natural sciences and SSH article titles substantially increases over the last 40 years (except of course for Mathematics, Physics, and Earth & Space). This suggests that title wording in scientific articles, at least in terms of vocabulary, becomes increasingly standardized over time. Needless to say, this lexical homogenization contrasts sharply with the cumulative picture of science given by [63], picture based on the expansion of the noun phrase lexica of physics, astronomy, and biomedicine article titles. While the present study is based on different linguistic entities (words instead of noun phrases), its results warrants a different picture of scientific entitling, according to which article titles get written using an increasingly restricted and cross-disciplinary set of words.

## Conclusion

The present research has proven fruitful on both algorithmic and empirical grounds. At the operational level, while both Zipf head and tail regimes outperform the other indicators used in the research, Zipfian distribution tail fits are more stable and robust than their head counterparts. In particular, their lower corpus size dependency proves extremely handy in distinguishing actual patterns from fluctuations revealed by other indicators and in representing artificial effects of corpus size fluctuations.

From an empirical perspective, the present research also shows that the lexical concentration of scholarly titles in Natural Sciences & Engineering and Social Sciences & Humanities articles increases by a little less than 8% over the [1975, 2014] period. At the disciplinary level, Mathematics, Earth & Space, and Physics titles have increased in lexical diversity to varying degrees, due probably and inter alia to the frequent use of eponymous nomenclature in these fundamental disciplines. Article titles from other natural sciences disciplines, which have a higher lexical diversity than those mentioned above, have all increased in lexical concentration over the observed period, which suggest a probable convergence of lexical concentration levels in the near future. With regard to Social Sciences & Humanities disciplines, lexical concentration levels remain surprisingly stable over the period, but aggregation effects observed at the disciplinary group level suggests that a cross-disciplinary homogenization of the highest word frequency ranks may be at work. Thus, with the exception of the most ancient natural sciences, title wording in scientific article titles becomes further standardized over time, as article titles get written using an increasingly restricted and cross-disciplinary set of words.

As a matter for future research, it would be interesting to correlate our new Zipf-based concentration indicator on bigger corpora like abstracts or full texts. The relationship between the concentration of word corpora and the concentration of article citations or citation parameters and patterns might also offer interesting insights. With regard to linguistic matters, the biggest issue fueled but left unaddressed by this article relates to explaining the prevalence of zipfian distributions in language. Indeed, given that “random texts do not exhibit the real Zipf’s law-like rank distribution,” [140] systematic and recurrent observation of zipfian patterns in natural languages suggests “that there is a “meaningful” mechanism at play.” [141] However, very little research has been done on this subject:

Essentially all of the work in language research has focused solely on deriving the law itself in principle; very little work has attempted to assess the underlying assumptions of the hypothesized explanation, a problem for much work on power laws in science [116].

Zipf himself interpreted his law as evidence for the ‘principle of least effort’ [104], which holds that the law-like form of frequency-rank distributions observed in natural languages results from an “evolutionary optimization process that minimizes some form of language usage cost.” [141] While Zipf did not provide “a clear logical development from this principle to action,” [107] a number of models were recently developed with the aim of generating zipfian distributions based on the principle of least effort [141, 142].

Mandelbrot explained for its part the law-like behaviour of word frequency-rank distributions using ideas from information theory [143]:

The essence of Mandelbrot’s contribution was his considering communication costs of words in terms of the letters that spell the words and the spaces that separate them. This cost increases [logarithmically] with the number of letters in a word and, by extension, in a message. Mandelbrot showed that Zipf’s law… follows as a first approximation from the minimization of communication costs in terms of letters and spaces. Linguistically, this amounts to minimizing costs in terms of phonemes, which is why the phenomenon holds for both written and spoken language [107].

Following Mandelbrot’s attempts, more recent models have shown how power laws can result from both logarithmic cost function minimization and entropy maximization [144]. A simpler hypothesis, straightforward yet providing a broader explanatory basis for zipfian distributions in language, can however be built from a structural isomorphism [116]. Following a series of experiments corroborating the idea that “memory mirrors… the structure that exists in the environment,” [145] Anderson hypothesizes that this goodness-of-fit of memory to environmental structure is of causal origin, in other words “that memory has the structure it has because the environment has the structure it has. [145]” Correspondingly, given the empirically validated omnipresence of zipfian structures in both the environment [146–148] and natural languages [116], it is reasonable to believe that natural languages present zipfian structures simply because the world they talk about and refer to is itself zipfian. In other words “Zipfian structures in the real world might ultimately create the observed form of word frequencies distributions.” [116]

While the present research has not provided any additional clue as to “which explanation, if any, is on the right track,” [116] analysis of frequency-rank distributions of scholarly article titles over the years has made one thing certain: “grabbing words by the tail” represents the best way found so far to accurately and reliably assess lexical diversity in language samples.

## Supporting information

S1 Appendix Note on the fitting procedure for Eq (9). (PDF)

S2 Appendix Annual lexical concentration scores for Natural Sciences & Engineering and Social Sciences & Humanities. All scores were normalized to the first plotted year (1975).
(PDF)

S3 Appendix Annual lexical concentration scores for Natural Sciences & Engineering disciplines. All scores were normalized to the first plotted year (1975).
(PDF)

S4 Appendix Annual lexical concentration scores for Social Sciences & Humanities disciplines. All scores were normalized to the first plotted year (1975).
(PDF)
