# Supplementary material for: Words by the tail: Assessing lexical diversity in scholarly titles using frequency-rank distribution tail fits
Source: PLoS One. 2018 Jul 9;13(7):e0197775. doi: 10.1371/journal.pone.0197775 (PMC6037356; doi:10.1371/journal.pone.0197775)
Supplement: S1 Appendix — (PDF) [file pone.0197775.s001.pdf]

The fitting of Equation 8 on data from word corpuses is not trivial, given that the empirical data is discrete and the function is continuous. For example, in a corpus of 100 word types in which 30 types occur only once in the corpus, the rank of all 30 single-occurring words is ambiguous. Such ambiguity, holding for every group of words of identical token count, results in the occurrence of plateaus and oscillations, such as in the Health tail plot shown in the S4 Appendix of the Supporting Information section.

We can answer this dilemma by comparing the ordered distribution function of a random sample of a corpus a fraction of the size to the ordered distribution function of the whole corpus where each occurrence is multiplied by the same fraction. The latter function is close to the ideal function for that random sample, save for the fact that the number of occurrences can be fractional. The following figure compares two frequency-rank distributions extracted from the 2014 WoS title word corpus, one based on the whole corpus an another on a 1/100th sample.

Partial and Complete Frequency-Rank distributions of the 2014 WoS title word corpus.

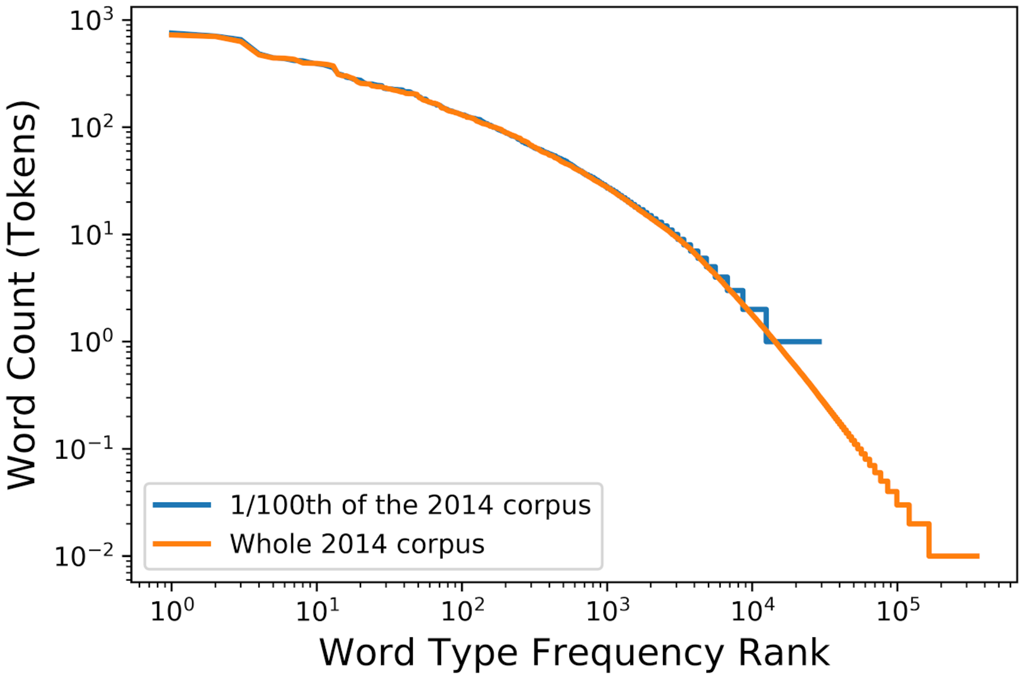

This figure shows that we should fit the curves to the leftmost point of each discrete plateau, with a weight proportional to the number of words in the plateau. This is equivalent to saying that, for our theoretical 100-words corpus, the rank for the 30 words that have only one occurrence is 70.

We can also solve this problem by randomly separating the corpus in half. One half would serve to attribute a word his rank, and the other would serve to attribute its number occurrences. This technique is used to ensure independence between the two parameters. This creates a disordered distribution function and gets rid of any plateaus in the curve. However, this process must be repeated until convergence, which can take dozens of repetitions. It is also unclear how to average all fitted parameters over each repetition when some parameters are multiplicative constants and others are exponents. There is also a greater chance of finding local minima in a disordered distribution function, and therefore more instability with the fitting process. This process is ideal, but since it is more complicated and would take much more time for very similar results, the former strategy was chosen here.
