# Supplementary figures and images for: Words by the tail: Assessing lexical diversity in scholarly titles using frequency-rank distribution tail fits
Source: PLoS One. 2018 Jul 9;13(7):e0197775. doi: 10.1371/journal.pone.0197775 (PMC6037356; doi:10.1371/journal.pone.0197775)

# NaturalSciences

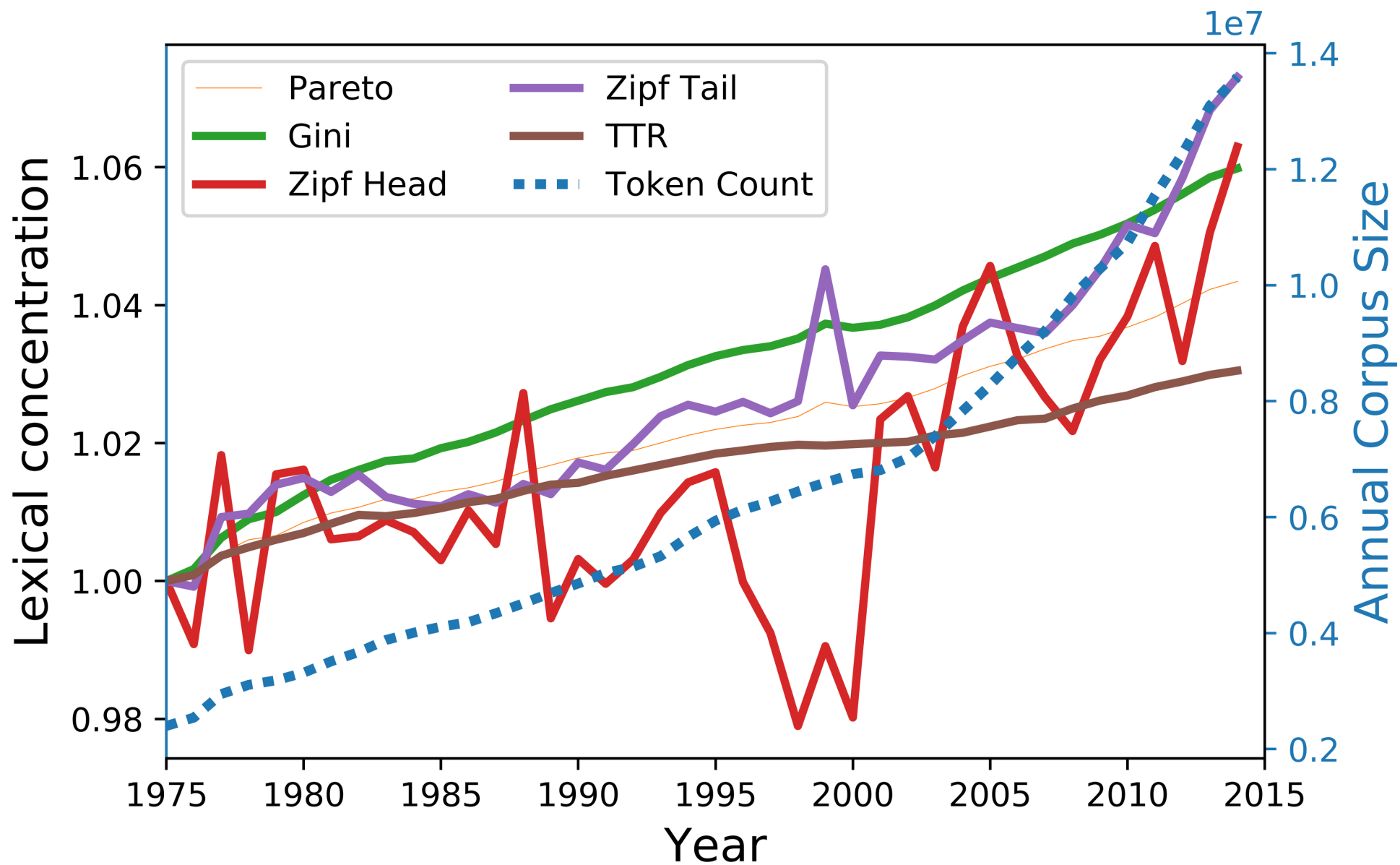

# SocialSciencesHumanities

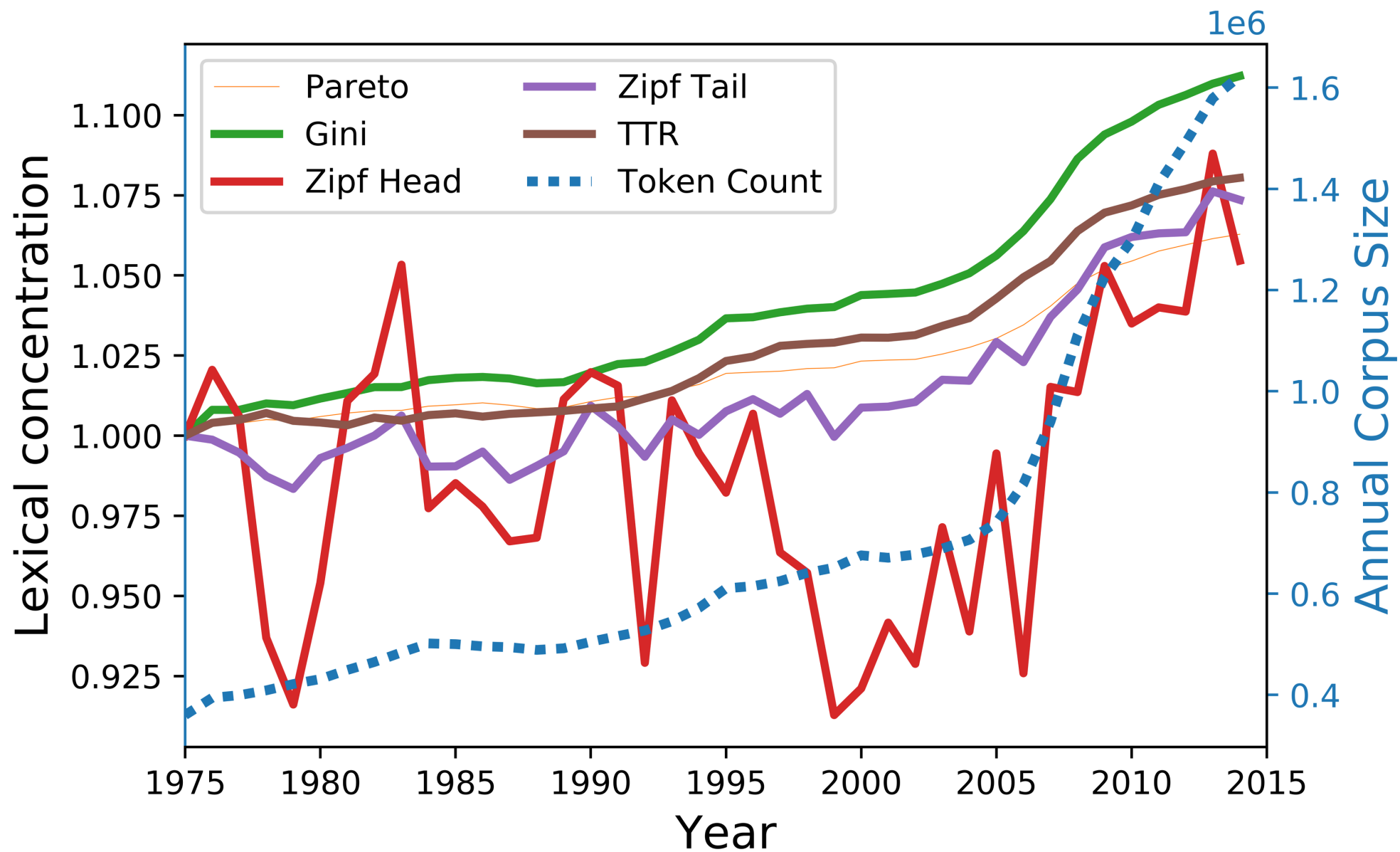

Supplement: S2 Appendix — All scores were normalized to the first plotted year (1975). (PDF) [file pone.0197775.s002.pdf]

# Health

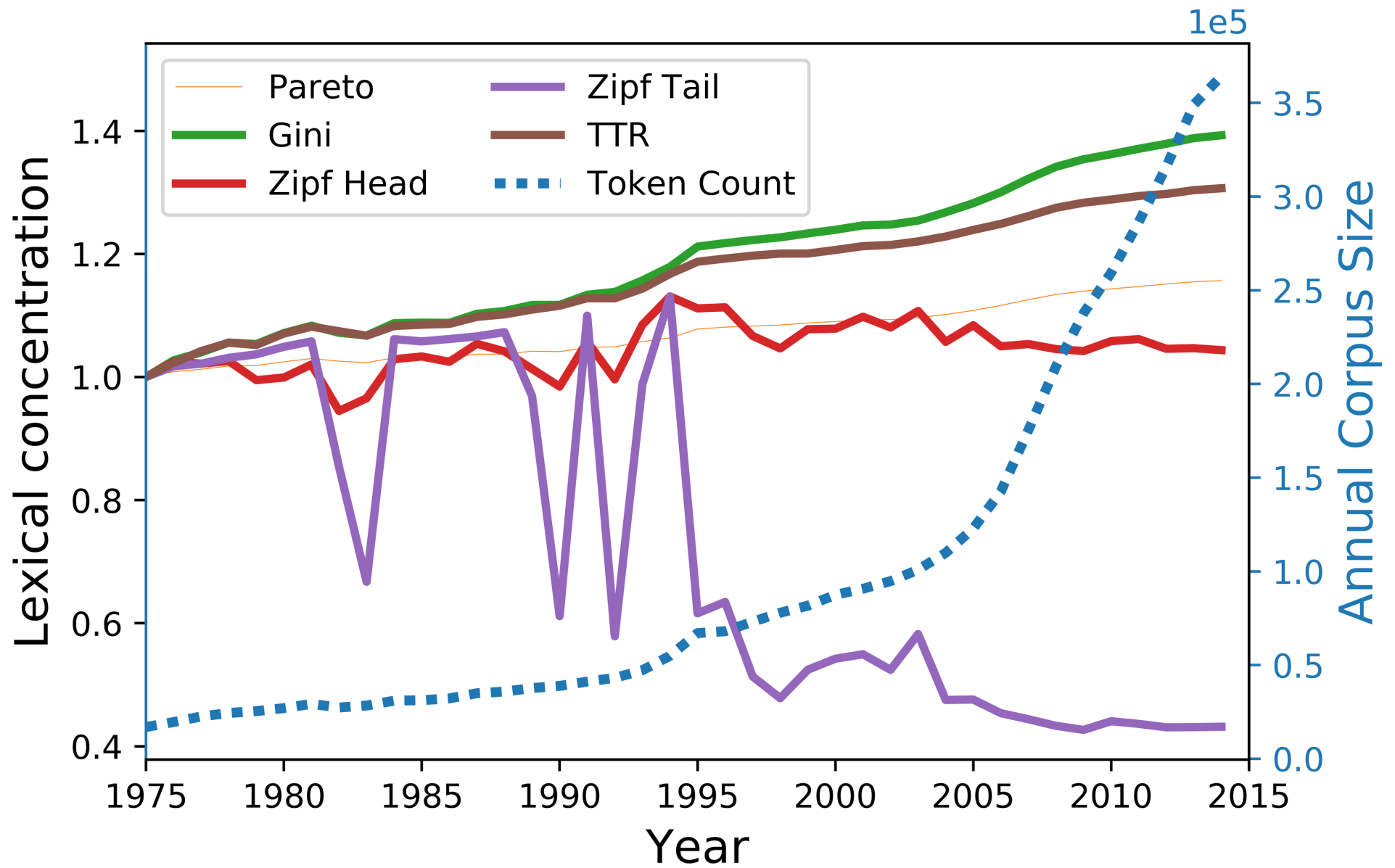

# Humanities

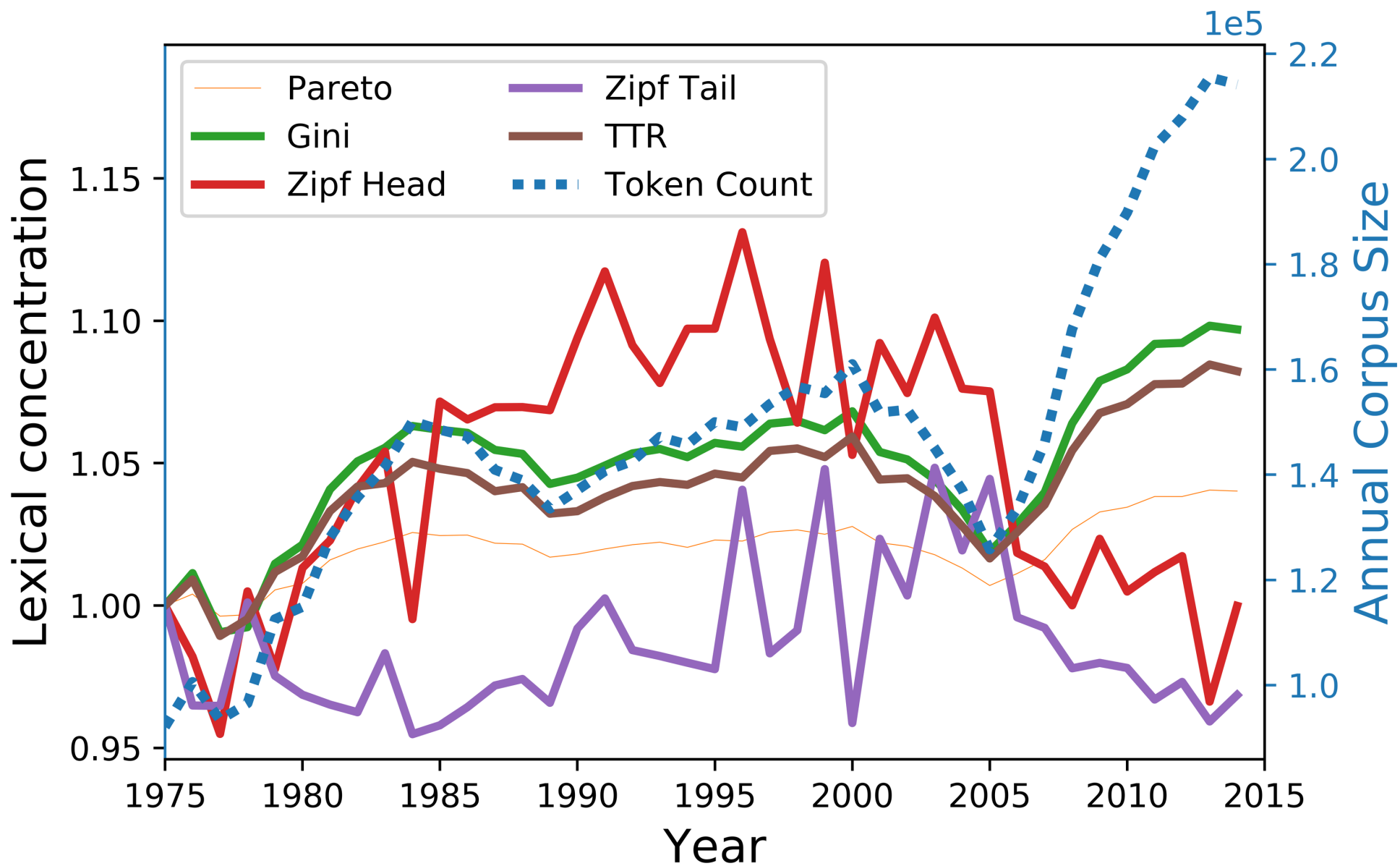

# Professional Fields

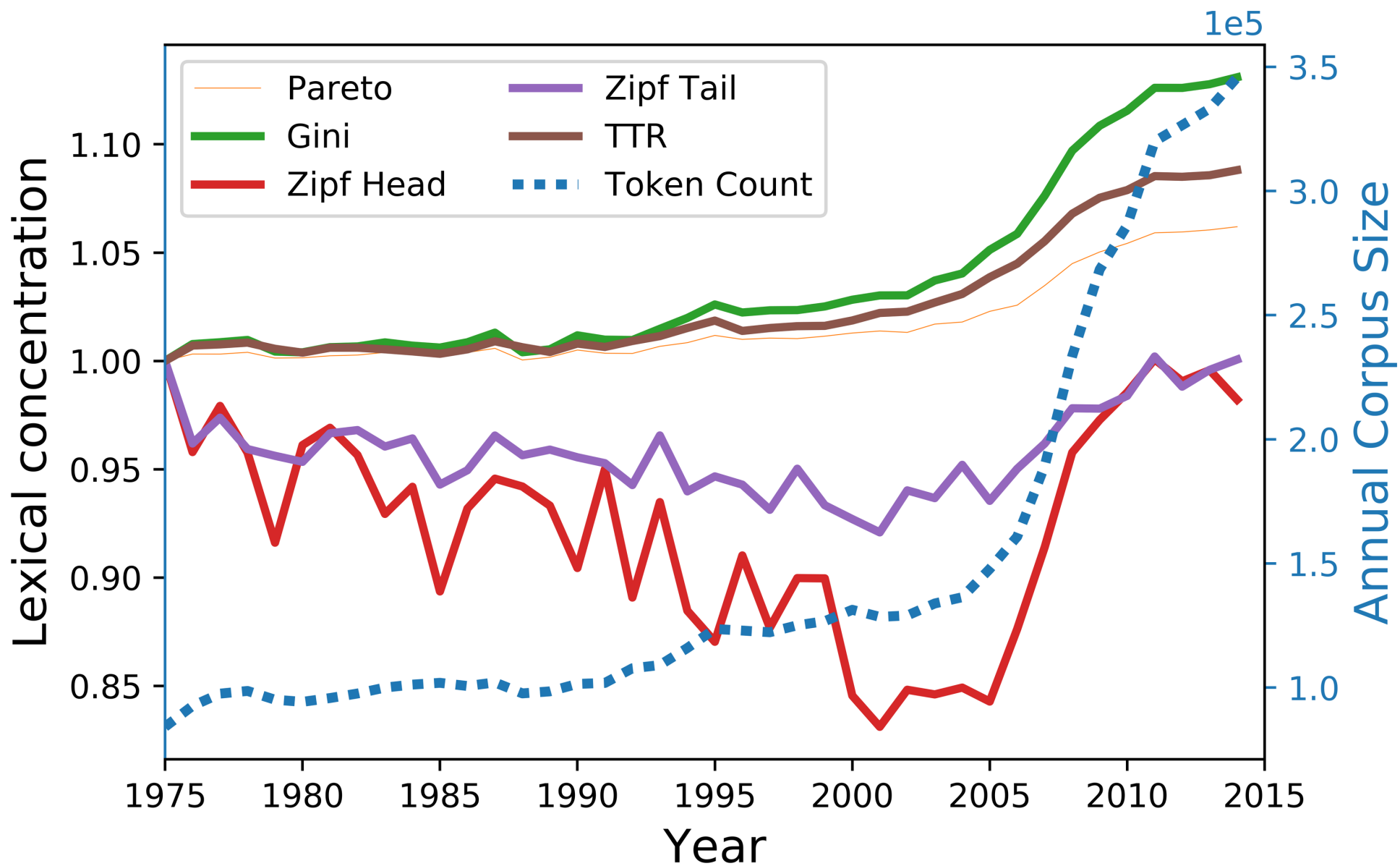

# Psychology

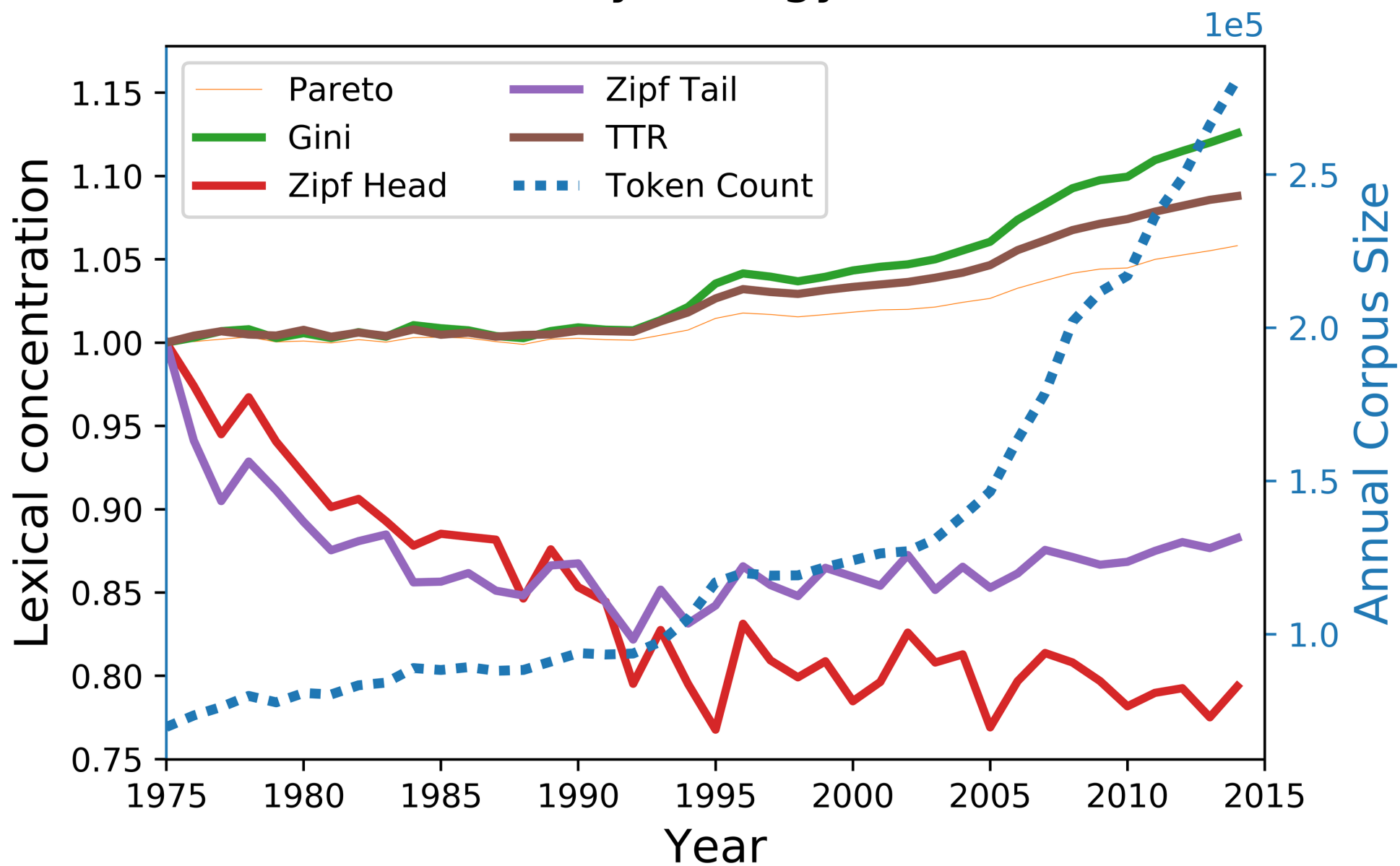

# Social Sciences

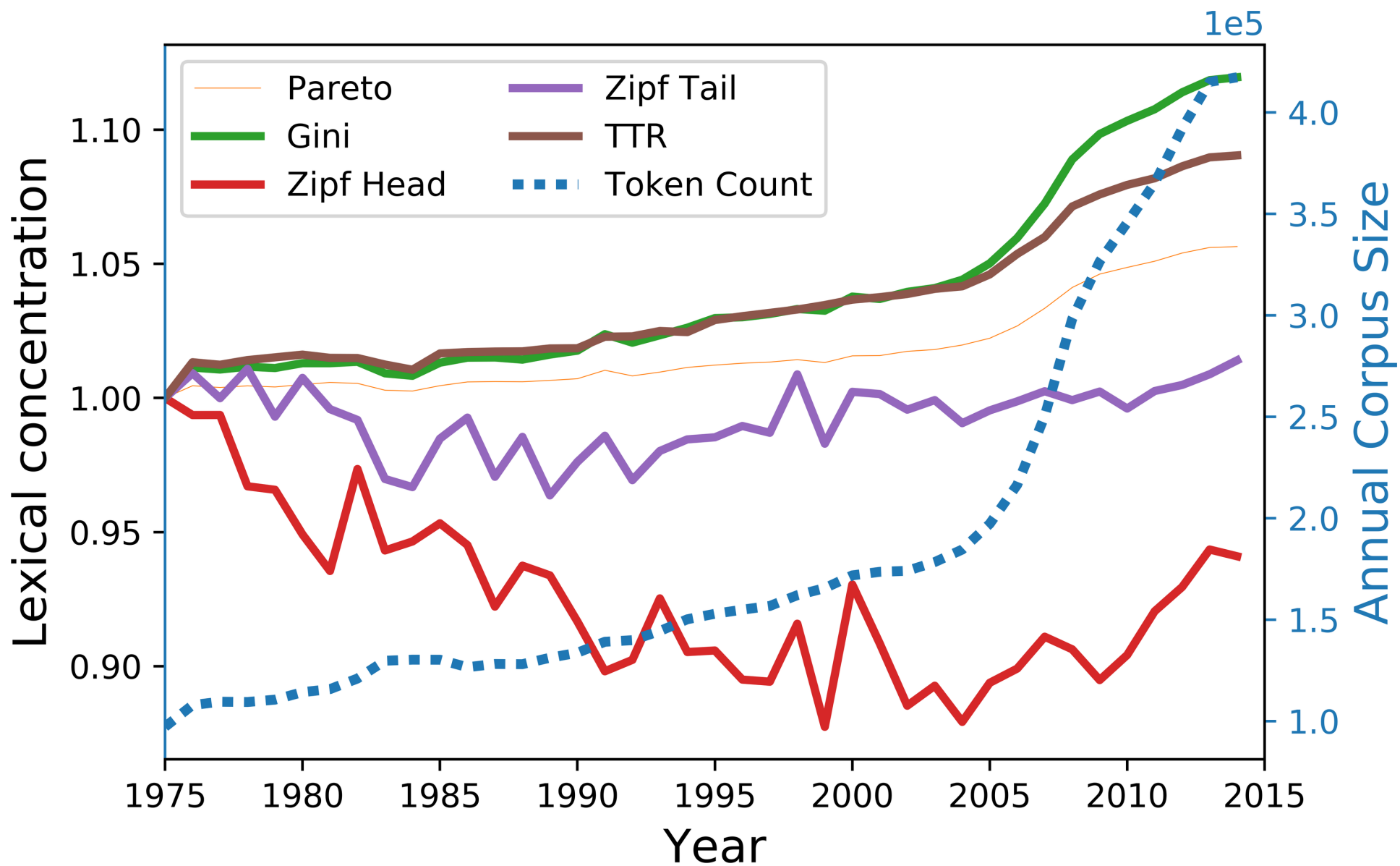

Supplement: S4 Appendix — All scores were normalized to the first plotted year (1975). (PDF) [file pone.0197775.s004.pdf]
