# Supplementary material for: Words by the tail: Assessing lexical diversity in scholarly titles using frequency-rank distribution tail fits
Source: PLoS One. 2018 Jul 9;13(7):e0197775. doi: 10.1371/journal.pone.0197775 (PMC6037356; doi:10.1371/journal.pone.0197775)
Supplement: S3 Appendix — All scores were normalized to the first plotted year (1975). (PDF) [file pone.0197775.s003.pdf]

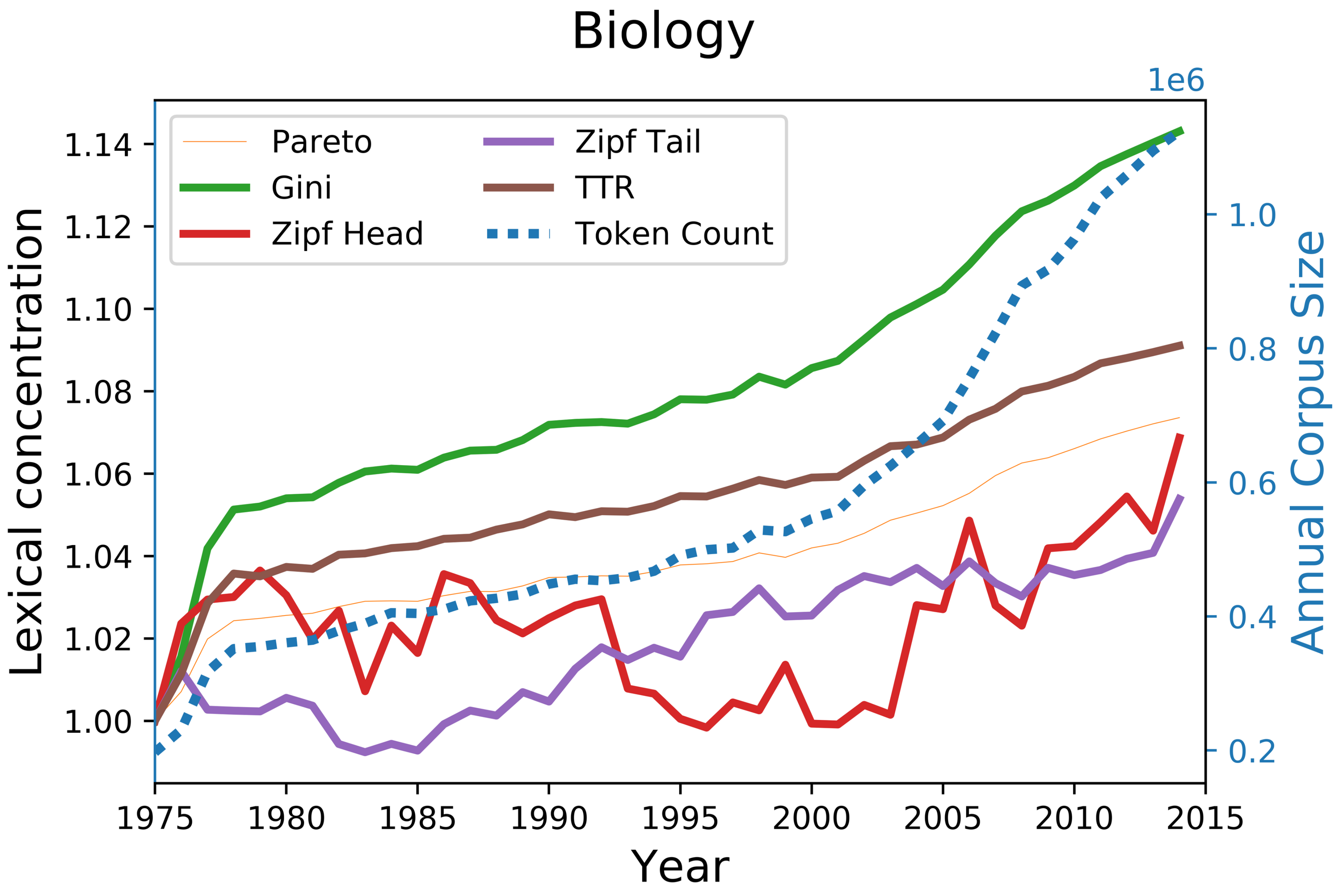

Biology
1e6
Pareto
Gini
Zipf Head
Zipf Tail
TTR
Token Count
Lexical concentration
Annual Corpus Size
Year
1.14
1.12
1.10
1.08
1.06
1.04
1.02
1.00
1.0
0.8
0.6
0.4
0.2
1975
1980
1985
1990
1995
2000
2005
2010
2015

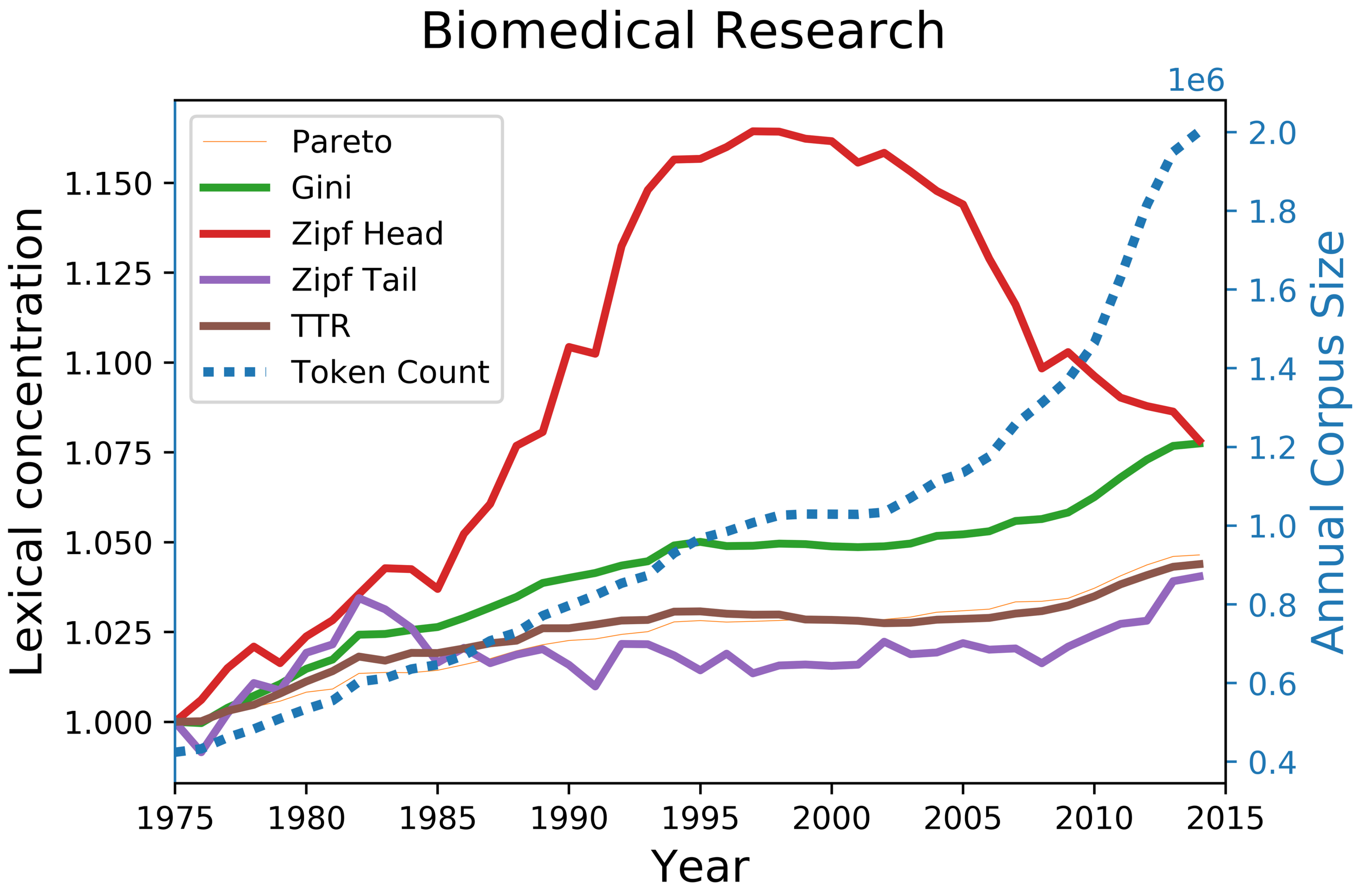

Biomedical Research
1e6
Pareto
Gini
Zipf Head
Zipf Tail
TTR
Token Count
Lexical concentration
1.150
1.125
1.100
1.075
1.050
1.025
1.000
Annual Corpus Size
2.0
1.8
1.6
1.4
1.2
1.0
0.8
0.6
0.4
1975
1980
1985
1990
1995
2000
2005
2010
2015
Year

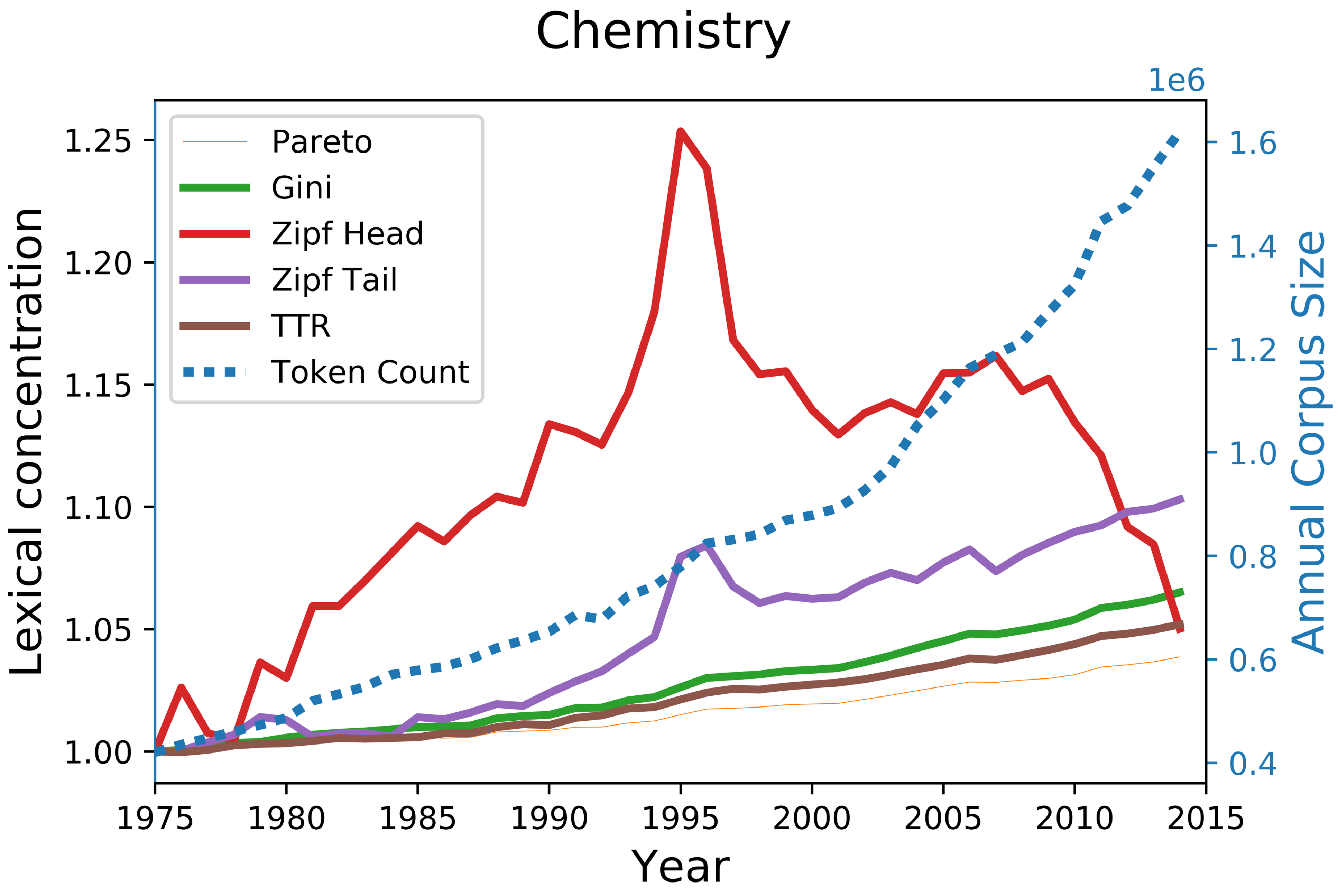

Chemistry
Lexical concentration
1.25
1.20
1.15
1.10
1.05
1.00
1e6
1.6
1.4
1.2
1.0
0.8
0.6
0.4
Annual Corpus Size
1975
1980
1985
1990
1995
2000
2005
2010
2015
Year
Pareto
Gini
Zipf Head
Zipf Tail
TTR
Token Count

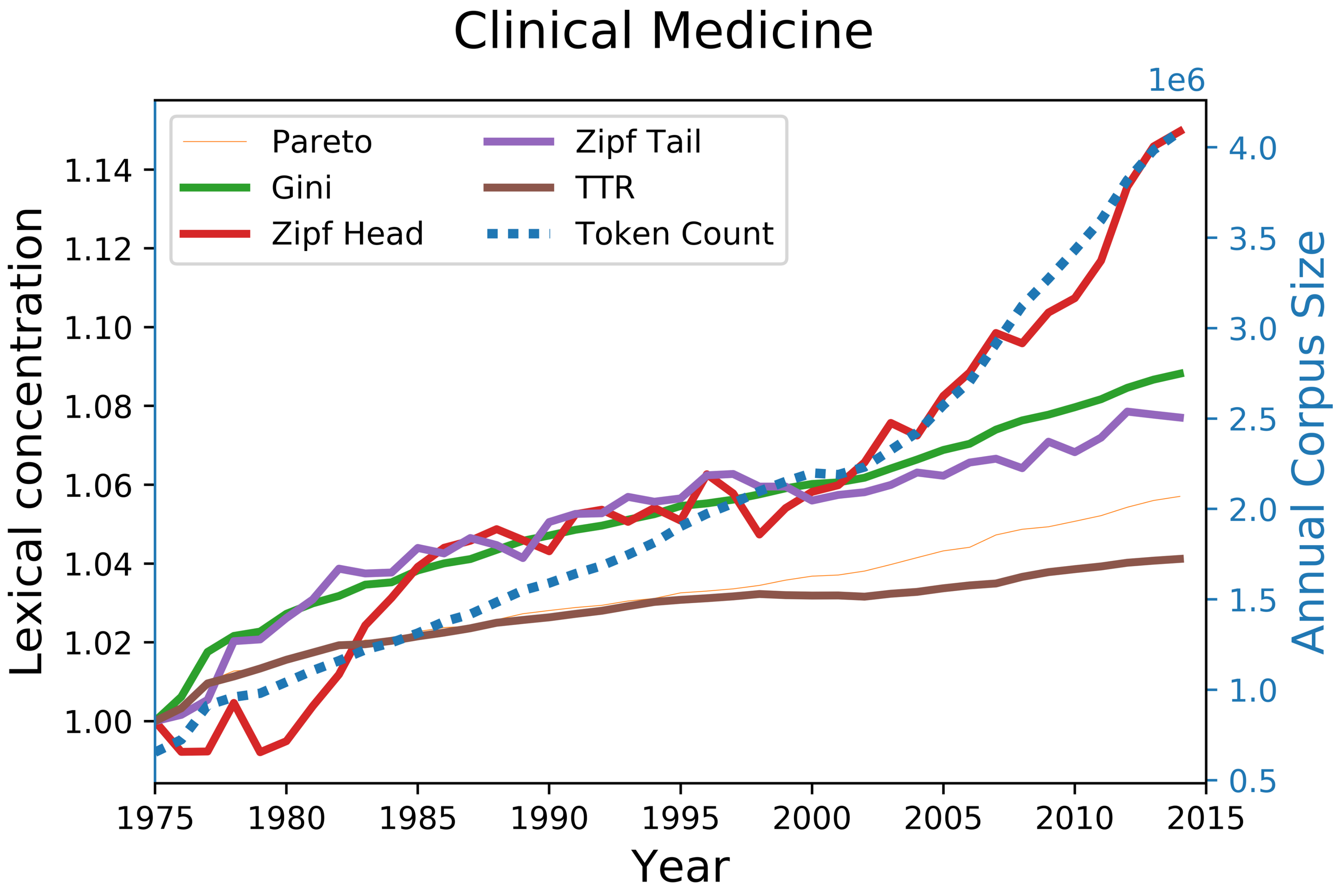

Clinical Medicine
1e6
Pareto
Gini
Zipf Head
Zipf Tail
TTR
Token Count
Lexical concentration
1.00
1.02
1.04
1.06
1.08
1.10
1.12
1.14
Annual Corpus Size
0.5
1.0
1.5
2.0
2.5
3.0
3.5
4.0
Year
1975
1980
1985
1990
1995
2000
2005
2010
2015

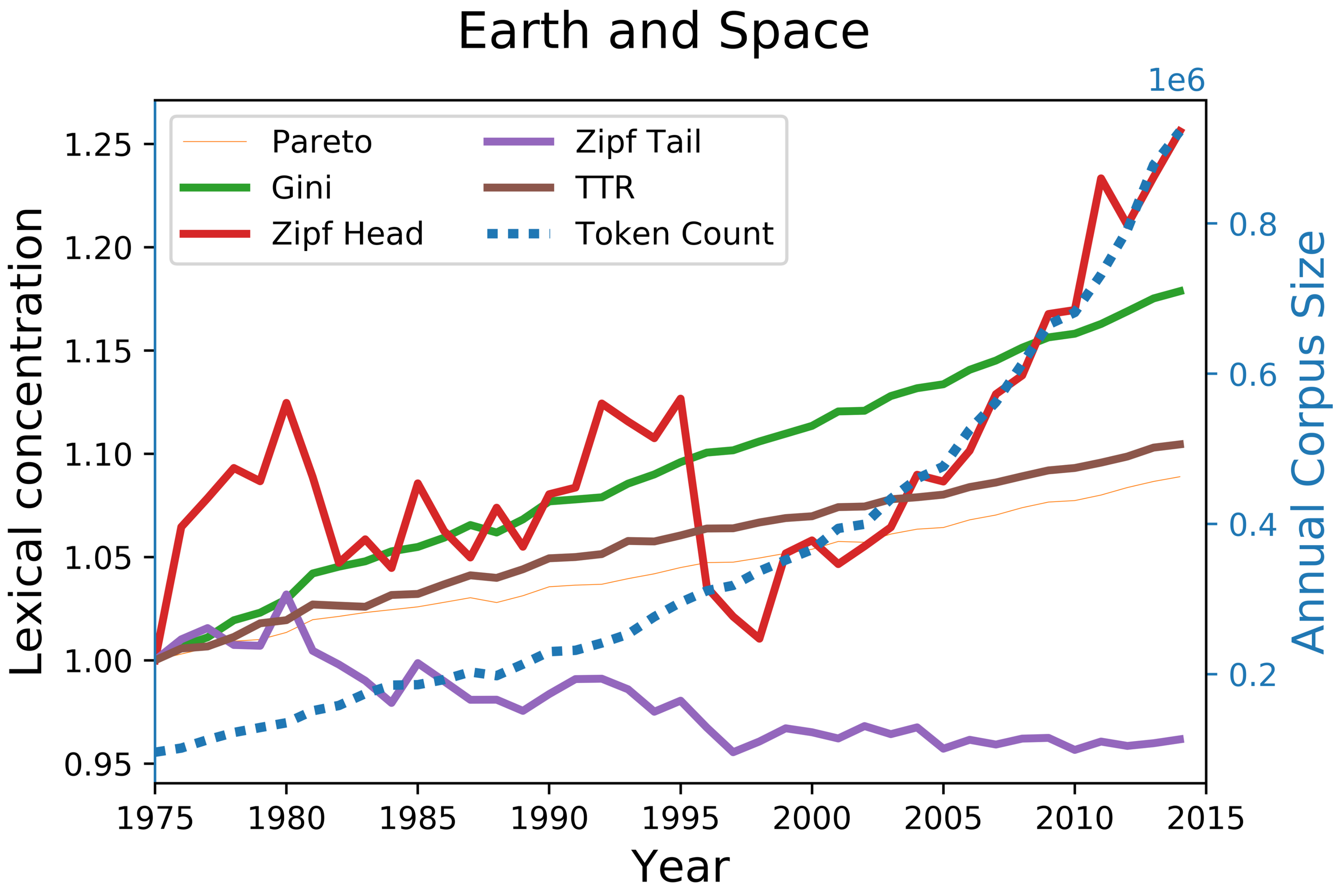

Earth and Space
1e6
Pareto
Gini
Zipf Head
Zipf Tail
TTR
Token Count
Lexical concentration
Annual Corpus Size
Year
1.25
1.20
1.15
1.10
1.05
1.00
0.95
0.8
0.6
0.4
0.2
1975
1980
1985
1990
1995
2000
2005
2010
2015

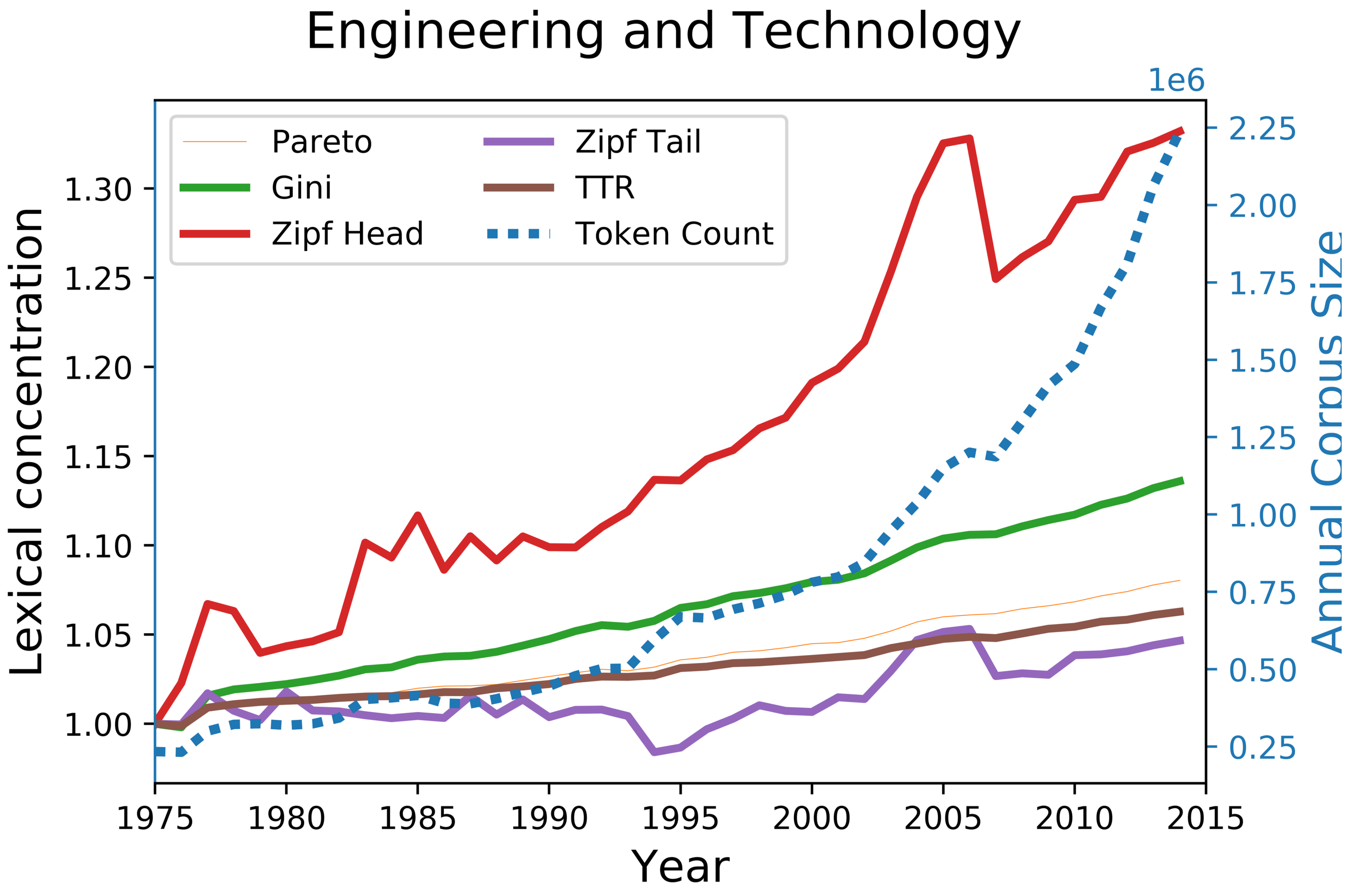
Engineering and Technology
1e6
Pareto
Gini
Zipf Head
Zipf Tail
TTR
Token Count
Lexical concentration
Annual Corpus Size
Year
1.00
1.05
1.10
1.15
1.20
1.25
1.30
0.25
0.50
0.75
1.00
1.25
1.50
1.75
2.00
2.25
1975
1980
1985
1990
1995
2000
2005
2010
2015

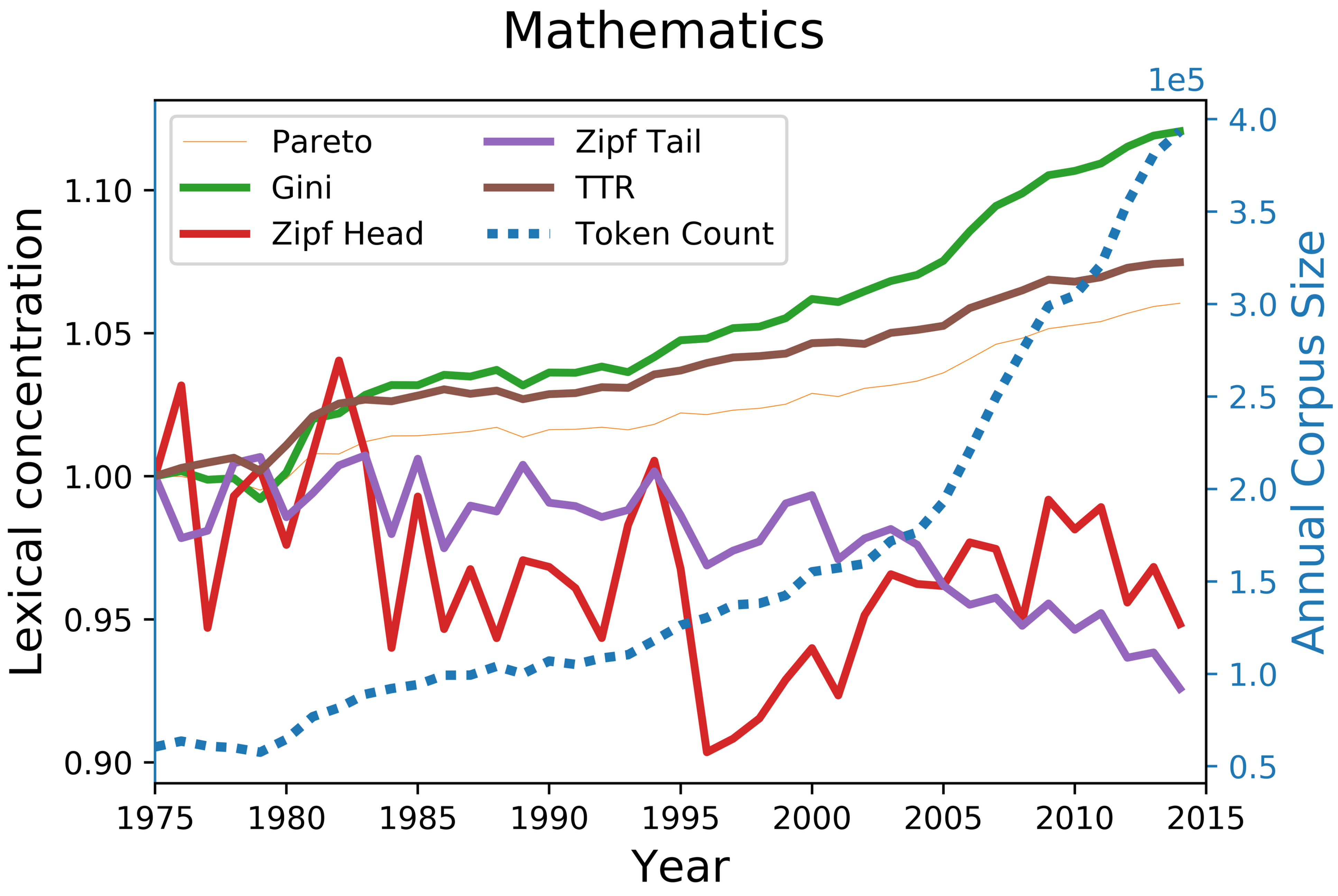

Mathematics
1e5
Pareto
Gini
Zipf Head
Zipf Tail
TTR
Token Count
Lexical concentration
Annual Corpus Size
Year
1.10
1.05
1.00
0.95
0.90
4.0
3.5
3.0
2.5
2.0
1.5
1.0
0.5
1975
1980
1985
1990
1995
2000
2005
2010
2015

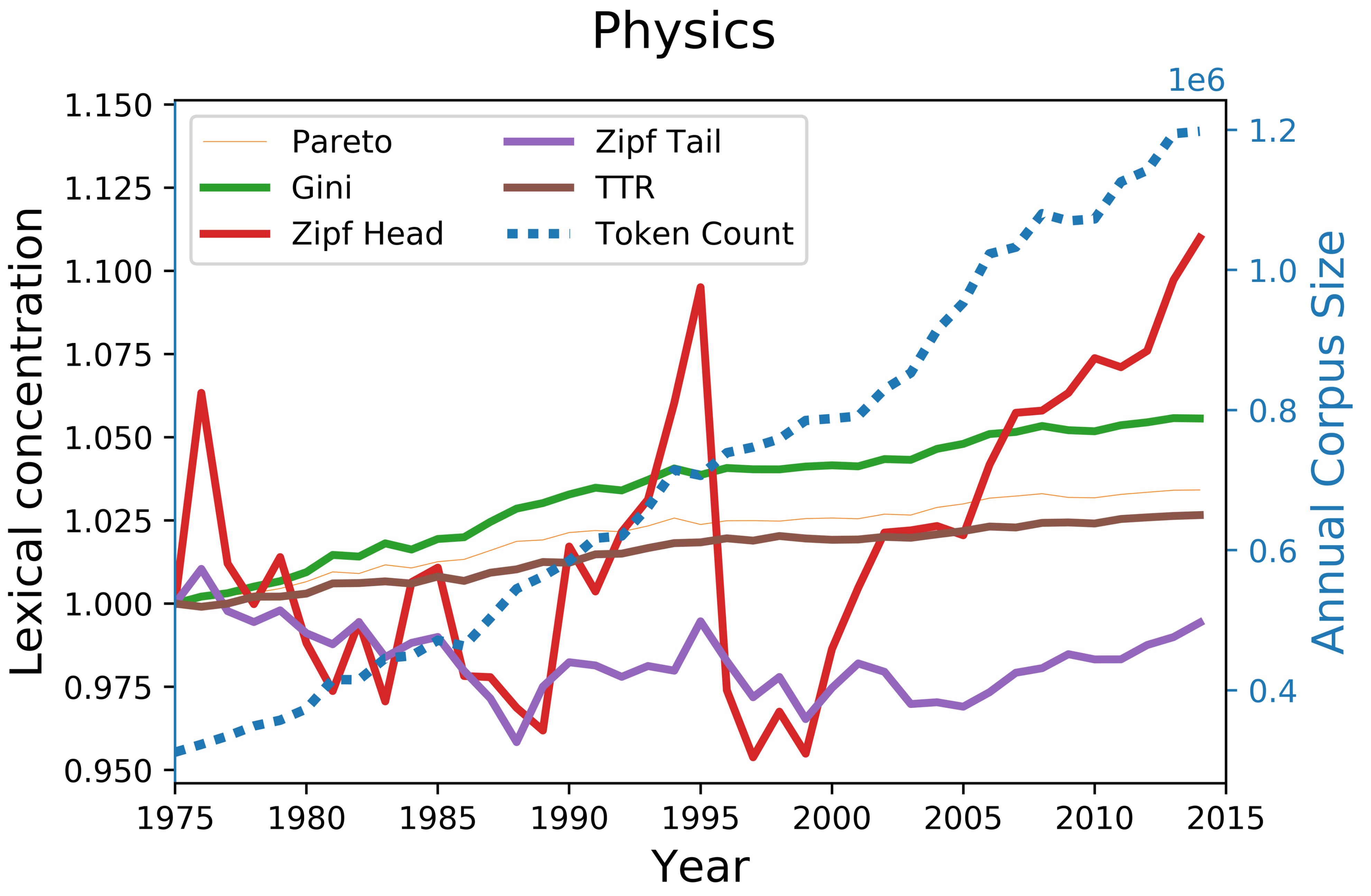
Physics
Pareto
Gini
Zipf Head
Zipf Tail
TTR
Token Count
Lexical concentration
Annual Corpus Size
1e6
Year
1.150
1.125
1.100
1.075
1.050
1.025
1.000
0.975
0.950
1.2
1.0
0.8
0.6
0.4
1975
1980
1985
1990
1995
2000
2005
2010
2015
